# The synaptic scaffold protein MPP2 interacts with GABA_A_ receptors at the periphery of the postsynaptic density of glutamatergic synapses

**DOI:** 10.1371/journal.pbio.3001503

**Published:** 2022-03-21

**Authors:** Bettina Schmerl, Niclas Gimber, Benno Kuropka, Alexander Stumpf, Jakob Rentsch, Stella-Amrei Kunde, Judith von Sivers, Helge Ewers, Dietmar Schmitz, Christian Freund, Jan Schmoranzer, Nils Rademacher, Sarah A. Shoichet

**Affiliations:** 1 Neuroscience Research Center, Charité–Universitätsmedizin Berlin, Germany; 2 Advanced Medical BioImaging Core Facility–AMBIO, Charité–Universitätsmedizin Berlin, Germany; 3 Institute of Chemistry and Biochemistry, Freie Universität Berlin, Germany; 4 German Center for Neurodegenerative Diseases (DZNE), Berlin, Germany; 5 Einstein Center for Neurosciences, Berlin, Germany; Thomas Jefferson University, UNITED STATES

## Abstract

Recent advances in imaging technology have highlighted that scaffold proteins and receptors are arranged in subsynaptic nanodomains. The synaptic membrane-associated guanylate kinase (MAGUK) scaffold protein membrane protein palmitoylated 2 (MPP2) is a component of α-amino-3-hydroxy-5-methyl-4-isoxazolepropionic acid (AMPA) receptor–associated protein complexes and also binds to the synaptic cell adhesion molecule SynCAM 1. Using superresolution imaging, we show that—like SynCAM 1—MPP2 is situated at the periphery of the postsynaptic density (PSD). In order to explore MPP2-associated protein complexes, we used a quantitative comparative proteomics approach and identified multiple γ-aminobutyric acid (GABA)_A_ receptor subunits among novel synaptic MPP2 interactors. In line with a scaffold function for MPP2 in the assembly and/or modulation of intact GABA_A_ receptors, manipulating MPP2 expression had effects on inhibitory synaptic transmission. We further show that GABA_A_ receptors are found together with MPP2 in a subset of dendritic spines and thus highlight MPP2 as a scaffold that serves as an adaptor molecule, linking peripheral synaptic elements critical for inhibitory regulation to central structures at the PSD of glutamatergic synapses.

## Abstract 2

This study shows that the MAGUK scaffold protein MPP2 is located at the periphery of postsynaptic densities in excitatory neurons, where it interacts with GABA-A receptors, thereby serving as a functional adaptor that links excitatory and inhibitory components of synaptic transmission at glutamatergic synapses.

## Introduction

At postsynaptic sites on glutamatergic neurons, a complex arrangement of transmembrane receptors, scaffold molecules, and regulatory proteins enables the coordinated regulation of synaptic transmission (for reviews, see [1–3]). Recent advances in imaging technology have highlighted that scaffold proteins and receptors are not distributed evenly throughout the postsynapse, but instead are arranged in subsynaptic nanodomains [4]. These nanodomains are regions in which specific proteins are present at higher concentrations than in the surrounding areas, and they are individually regulated, functional units that are highly dynamic [5,6]. The relevance of their regulation for synaptic function is becoming increasingly apparent (for reviews, see [7–9]). Superresolution imaging data from glutamatergic synapses suggest that such scaffold protein nanoclusters are responsible for concentrating glutamate receptors at particular subsynaptic sites [5]. The incidence of these clusters seems to roughly scale with spine size, and clusters have been observed to undergo morphological plasticity [5,10]. Subsynaptic cluster dynamics at the postsynapse can also influence synaptic transmission, e.g., by affecting diffusion and/or trapping of neurotransmitter receptors [5,6,11].

We and others have recently demonstrated that membrane protein palmitoylated 2 (MPP2) is a postsynaptic scaffold protein that is present in α-amino-3-hydroxy-5-methyl-4-isoxazolepropionic acid (AMPA) receptor–associated protein complexes [12–15]. Like postsynaptic density (PSD)-95 and related molecules, MPP family proteins are members of the membrane-associated guanylate kinase (MAGUK) family of scaffold molecules. Different from PSD-95 molecules, they do not bind directly to glutamate receptors or their auxiliary subunits. However, MPP2 binds directly to SynCAM 1 synaptic cell adhesion molecules [13] that are positioned at the periphery of the PSD, suggesting that they may serve functions that differ from those of the core synaptic MAGUKs.

In this study, we have investigated this new postsynaptic MAGUK and its structural role at the PSD of glutamatergic synapses. We demonstrate that MPP2, like SynCAM 1, sits at the periphery of the PSD and that it interacts with a unique set of proteins that differs significantly in composition from the set of PSD proteins binding to PSD-95 family synaptic MAGUKs. These novel interactions highlight the role of MPP2 in linking core synaptic components to transmembrane proteins and regulatory molecules with complementary functions at the periphery of the PSD. Importantly, we show that multiple γ-aminobutyric acid (GABA)_A_ receptor subunits are among the proteins that bind most strongly to the MPP2 scaffold molecule. We show that GABA_A_ receptors are found together with MPP2 and other classical PSD markers in a subset of dendritic spines, and in line with a physical interaction between MPP2 and such GABA_A_ receptors, we observe an increased amplitude of miniature inhibitory postsynaptic currents (mIPSCs) following overexpression of MPP2 and a corresponding reduced mIPSC amplitude upon knockdown of MPP2. Moreover, we highlight that MPP2 expression does not overlap substantially with Gephyrin, the classical scaffold protein at inhibitory postsynapses. We thus demonstrate that the MPP2 scaffold molecule is capable of acting as an adaptor molecule that links important PSD protein complexes with elements critical for inhibitory regulation specifically at the PSD of glutamatergic synapses.

## Results

### MPP2 and its interaction partner SynCAM 1 are positioned at the periphery of the PSD

**Fig 1:**
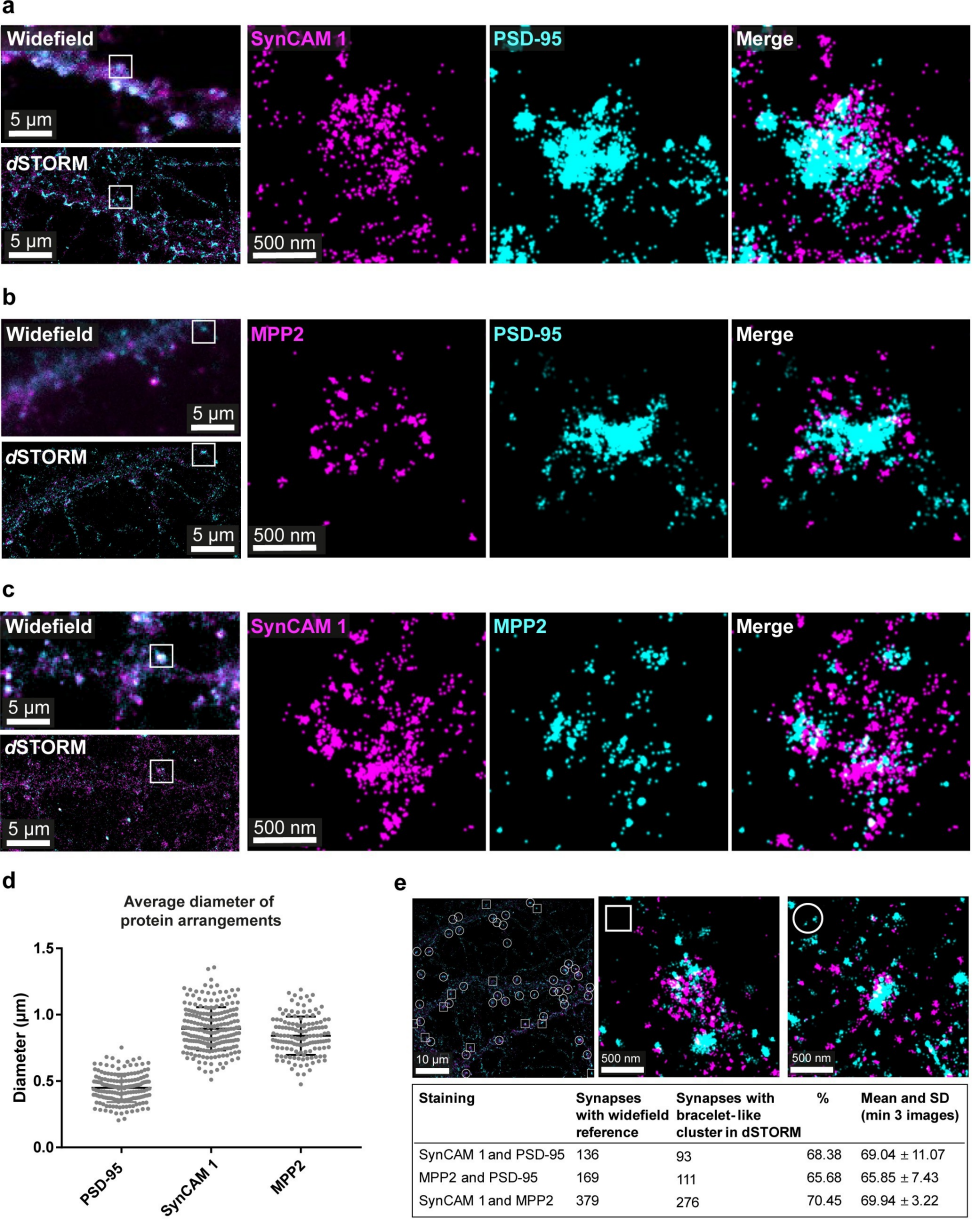
Clusters of SynCAM 1 and MPP2 surround the PSD. E18 rat hippocampal neurons were fixed at DIV21 and subjected to immunostaining for endogenous SynCAM 1, PSD-95, and/or MPP2 proteins followed by dual-colour *d*STORM imaging. Localisations were filtered according to precision estimates based on the Thompson method, i.e., all localisations with localisation precision worse than 20 nm were excluded. Localisations were rendered as Gaussian distributions with a constant width of 20 nm. **(a)** Corresponding widefield (left, top) and *d*STORM (left, bottom) images of a neuronal dendrite stained for endogenous SynCAM 1 and PSD-95. White box indicates location of the detail view of the individual synapse enlarged in the right panels. Clusters of SynCAM 1 (magenta) are arranged in a bracelet-like fashion surrounding postsynaptic densities marked by PSD-95 (cyan). **(b)** Corresponding widefield (left, top) and *d*STORM (left, bottom) images of a dendrite segment stained for endogenous MPP2 and PSD-95. White box indicates location of the synapse shown in the detail view (right panel). Clusters of endogenous MPP2 (magenta) show a similar bracelet-like arrangement at the periphery of the PSD (PSD-95, cyan). **(c)** Corresponding widefield (left, top) and *d*STORM (left, bottom) images of a neuronal dendrite stained for endogenous SynCAM 1 and MPP2. The white box indicates the location of the detail view of the individual synapse enlarged in the right panels. Adjacent protein clusters of SynCAM 1 (magenta) and MPP2 (cyan) form a bracelet-like arrangement at postsynaptic sites. For additional images, see Supporting information figures (S1–S3 Figs). Scale bars: overview 5 μm; detail 1 μm. **(d)** Average diameter of the observed protein arrangements of PSD-95, SynCAM 1 and MPP2. Included are measurements of 170 to 236 synapses in 6 to 8 images, from 2 to 4 independent experiments. Underlying data can be found in S2 Data. **(e)** Prevalence of bracelet-like arrangements of SynCAM 1 and MPP2 clusters surrounding PSDs. For more details on the process, please see also S4 Fig. Short, synapses were identified in corresponding widefield and *d*STORM images and manually assessed with regard to whether SynCAM 1 and MPP2 showed bracelet-like arrangements (circle) or not (square). Quantification is of 3 to 6 images per staining combination, from 2 to 3 independent experiments. DIV, days in vitro; *d*STORM, direct stochastic optical reconstruction microscopy; MPP2, membrane protein palmitoylated 2; PSD, postsynaptic density.

To investigate how MPP2 links the SynCAM 1 cell adhesion molecules with the core PSD components, we first took advantage of diverse imaging strategies to comparatively analyse endogenous PSD-95, MPP2, and SynCAM 1 proteins in primary rat hippocampal neurons. Using dual-colour direct stochastic optical reconstruction microscopy (*d*STORM), we found that in neurons expressing both SynCAM 1 and MPP2, several synapses exhibit an interesting arrangement of proteins, where neighbouring clusters of MPP2 and SynCAM 1 seem to form a bracelet-like structure around the central PSD marker PSD-95. The observed clusters of SynCAM 1 formed a bracelet-like arrangement of 894 nm (SD: 162 nm) diameter (Fig 1A, magenta and Fig 1D) surrounding the PSD marked by PSD-95 (Fig 1A, cyan and Fig 1D, diameter 448 nm, SD: 105 nm), which is in line with published data [16].

Next, we examined the subcellular localisation of endogenous MPP2 (Fig 1B, magenta) and found a similar bracelet-like arrangement (Fig 1D, diameter 791 nm, SD: 209 nm), of small clusters of MPP2 surrounding postsynaptic densities as marked by PSD-95 (Fig 1B, cyan). Further, when we stained for SynCAM 1 in combination with MPP2 (Fig 1C), we found that the 2 proteins are indeed arranged in a similar manner: We observed bracelet-like arrangements of SynCAM 1 (Fig 1C, magenta) and MPP2 (Fig 1C, cyan) clusters that tightly associate with each other and exhibit minor overlap. Not all synapses exhibited this bracelet-like arrangement of MPP2 and SynCAM 1 around the central PSD-95; we estimated their prevalence at 65% (Fig 1E).

### MPP2 is located in clusters at the periphery of the PSD

**Fig 2:**
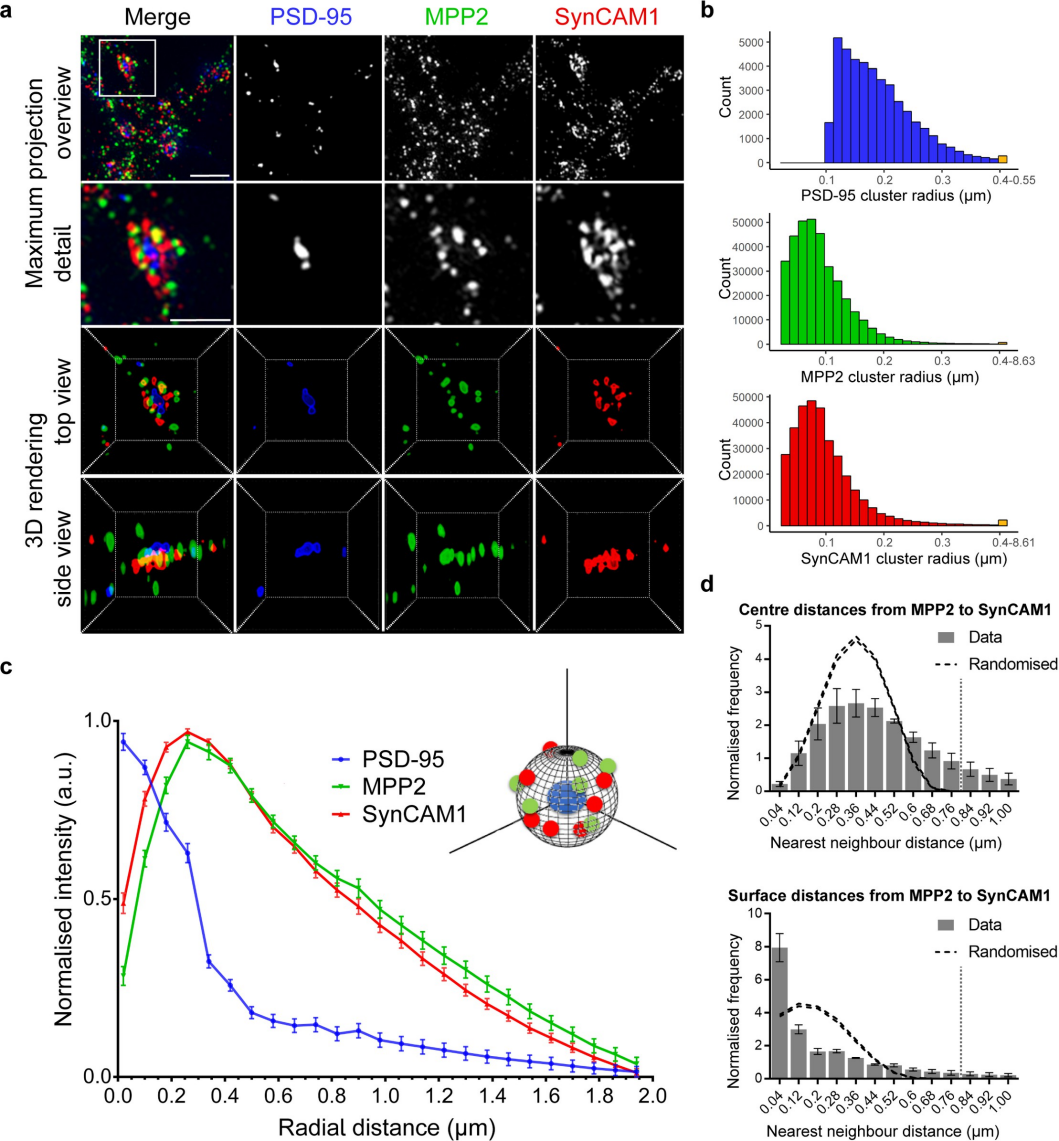
Clusters of MPP2 and SynCAM 1 form bracelet-like arrangements at the edge of the PSD. Mature (DIV21) primary rat hippocampal neurons immunostained for endogenous PSD-95 (blue, second column), MPP2 (green, third column), and SynCAM 1 (red, fourth column) and subjected to 3D SIM. **(a)** More than 90% of the imaged dendritic spines express all 3 proteins of interest (overview maximum projection, first row). A single synapse detail (second row) depicts the bracelet-like arrangements of MPP2 and SynCAM 1 surrounding central PSD-95 puncta. A 3D rendering of that particular synapse in top (third row) and side view (fourth row) reveals that SynCAM 1 and MPP2 clusters are arranged in an interlocked, bracelet-like form, surrounding a central cluster of PSD-95. Scale bars: overview 1 μm; detail 1 μm. Three-dimensional rendering box size: 2.8 μm. **(b)** Histograms illustrating the distribution of protein cluster sizes for PSD-95 (top, blue), MPP2 (middle, green), and SynCAM 1 (bottom, red). Indicated radii were calculated based on extracted cluster volumes, assuming a spherical shape. The final bin in each histogram contains summarised data for cluster sizes greater than 400 nm. Histograms reflect clusters associated with approximately 40,000 synapses (in 50 images from *N* = 3 independent experiments). **(c)** Three-dimensional radial intensity profiles of PSD-95, MPP2, and SynCAM 1 signals in relation to the centres of PSD-95 clusters. Plot shows averaged normalised mean ± SEM from 3 independent experiments (approximately 40,000 synapses from 50 images). For details on the analysis, please see the Materials and methods. **(d)** NN analysis of MPP2 and SynCAM 1 protein clusters after 3D segmentation. NN distances from MPP2 to the nearest SynCAM 1 cluster were calculated between cluster centres (upper panel, grey bars) and cluster surfaces (lower panel). Dashed lines represent the upper and lower envelopes of CSR. CSR was calculated by randomly distributing MPP2 within the volume and SynCAM 1 on the surface of spheres of 0.8 μm diameter as indicated by the grey dotted line (mean ± SEM, 95% confidence interval, 10 simulations per synapse, *N* = 3 independent experiments, approximately 40,000 synapses from 50 images). See S6 Fig for NN analysis in the reverse direction. Underlying data are provided in S2 Data. CSR, complete spatial randomness; DIV, days in vitro; MPP2, membrane protein palmitoylated 2; NN, nearest neighbour; PSD, postsynaptic density; SIM, structured illumination microscopy.

To assess whether this bracelet-like arrangement of MPP2 and SynCAM 1 clusters surrounding PSD-95 is representative for the majority of synapses (and to avoid selection bias), a quantitative 3D superresolution approach is necessary. We therefore tested whether we could resolve bracelet-like MPP2 and SynCAM 1 structures also using 3D multicolour structured illumination microscopy (3D SIM). While offering less spatial resolution compared to *d*STORM, SIM inherently produces 3D data and can easily provide 4 colour channels. Indeed, 3D SIM imaging was sufficient to observe similar planar bracelet-like cluster arrangements of SynCAM 1 and MPP2 surrounding central PSD-95 labelled PSDs (Fig 2A).

Using a semiautomated image segmentation pipeline (see Materials and methods), we quantitatively assessed the segmented object counts and radii as derived from the cluster volumes and found that more than 90% of the imaged synapses expressed all 3 proteins of interest. In line with published data [6,10] and our *d*STORM results, we found PSD-95 clusters over a range of expected sizes (Fig 2B, upper panel, blue). Interestingly, the majority of MPP2 (Fig 2B, middle panel, green) and SynCAM 1 (Fig 2B, lower panel, red) clusters are smaller than approximately 100 nm in radius.

Next, we quantitatively analysed the SynCAM 1 and MPP2 protein distribution in relation to PSD-95 clusters, by measuring 3D radial distribution of all 3 proteins around the PSD centre defined by the PSD-95 signal. The 3D radial intensity profile of PSD-95 immunofluorescence intensity drops considerably at a radial distance of approximately 250 nm (Fig 2C, blue curve), which is consistent with reported PSD sizes and our dual-colour *d*STORM data. The SynCAM 1 signal (Fig 2C, red curve) is low at the centre of the PSD and highest towards the border of the PSD (radial distance of approximately 250 nm), which is observable in the steep decrease in PSD-95 signal. These data are in line with the idea that clusters of SynCAM 1 define the edge of the PSD and the synaptic cleft [16]. Interestingly, the 3D radial intensity profile for MPP2 is almost identical to that of SynCAM 1: We observe little fluorescence towards the centre of the PSD (radial distances below 250 nm) and the highest signal at the PSD border (Fig 2C, green curve). This quantitative result validates our qualitative superresolution observation that MPP2, like SynCAM 1, is distributed at the periphery of the PSD, around the core PSD protein PSD-95.

To assess the spatial relationship of the peripheral SynCAM 1 and MPP2 protein clusters, we performed nearest neighbour (NN) analysis, which interrogates the nanoscale distances of the closest SynCAM 1 cluster to each MPP2 cluster and vice versa. This NN analysis was performed by assessing both the distances from centre to centre and from surface to surface for each 3D object. The distance distributions were quantitatively compared to a simulated random distribution within the known volume of a postsynapse [17]. The centre-to-centre NN analysis showed that most centres of MPP2 clusters have an NN distance of 200 nm to 500 nm to centres of SynCAM 1 clusters (Fig 2D, upper panel). Similar results were obtained when analysing the centre-to-centre distances of SynCAM 1 clusters to the nearest MPP2 cluster (see S5 Fig), showing that the 2 proteins do not form one uniform cluster. The obtained range of NN distances rather corresponds to the sum of both cluster radii (average cluster sizes are below 200 nm; Fig 2B, middle and lower panel), suggesting a juxtapose association of the proteins.

To test whether the surfaces of MPP2 and SynCAM 1 clusters overlap with each other, we performed a surface-to-surface NN analysis, which showed a significant accumulation of MPP2 clusters at very small distances to SynCAM 1 clusters and vice versa (Fig 2D, lower panel). This accumulation is very prominent regarding the MPP2 clusters that are located around SynCAM 1 clusters (Fig 2D, lower panel; note the significant increase over random of the first bin); however, it is less prominent when analysed inversely (see S6 Fig). This indicates that most MPP2 clusters are associated with SynCAM 1 clusters but not vice versa, suggesting the existence of an additional SynCAM 1 pool that is independent from the observed postsynaptic MPP2, which is in line with the fact that SynCAM 1 is also present at presynaptic sites. These data show that MPP2 and SynCAM 1 clusters are not completely overlapping, but tightly juxtaposed at the periphery of the PSD.

Although clusters of SynCAM 1 and MPP2 are not spatially identical, their close association offers sufficient chances for molecular interaction with each other. In summary, our superresolution data provide evidence that MPP2 and SynCAM 1 are postsynaptic proteins in a close subsynaptic arrangement sitting at the periphery of postsynaptic densities and thus highlight the potential for the scaffold protein MPP2 to mediate formation of complexes that are distinct from the central nanodomains orchestrated by the neighbouring MAGUK PSD-95.

### The carboxyl-terminal SH3GK domains of MPP2 and PSD-95 interact with distinct synaptic proteins

**Fig 3:**
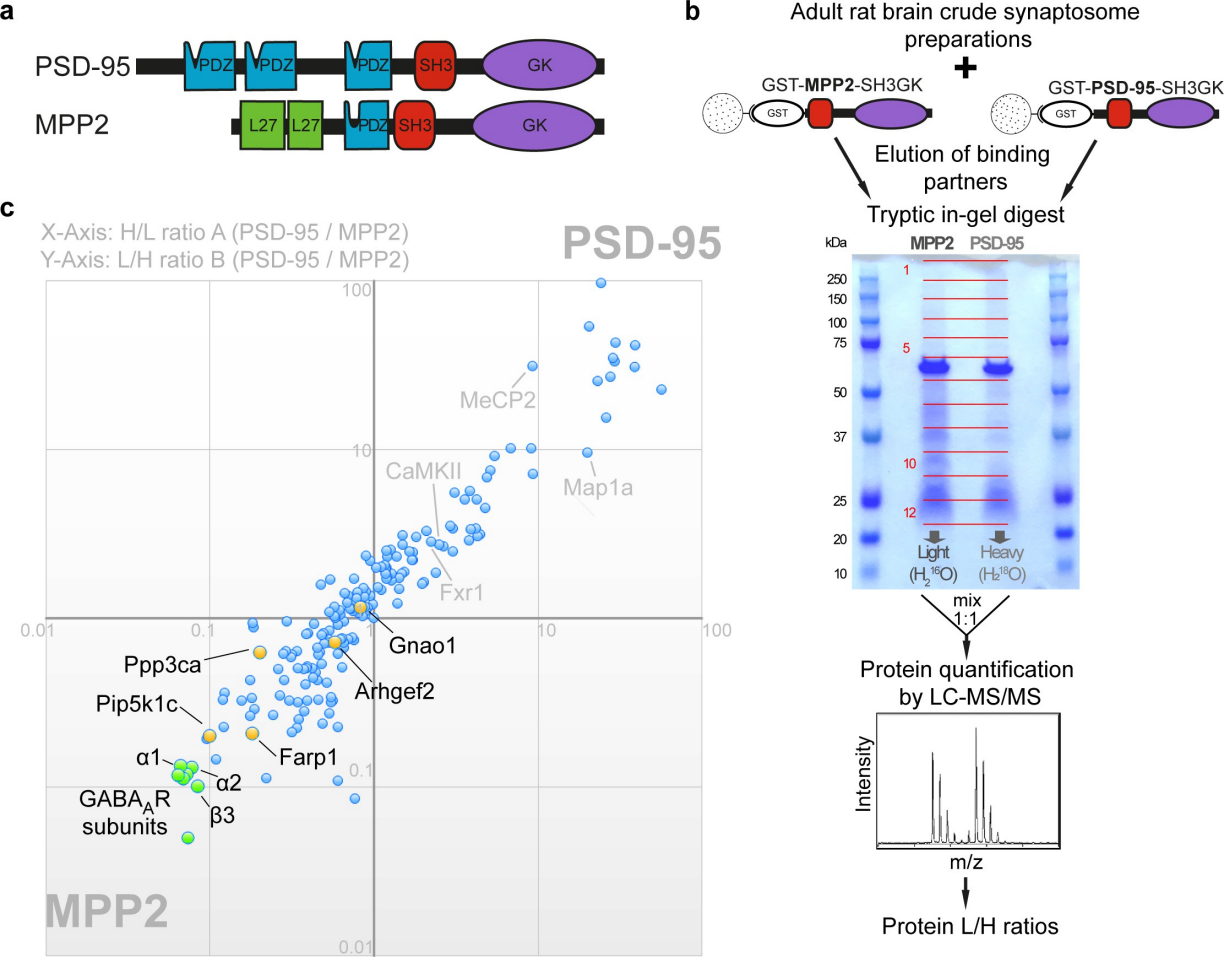
Identification of interactors that differentially bind to the carboxyl-terminal SH3GK modules of MPP2 and/or PSD-95. **(a)** Schematic domain structures of PSD-95 and MPP2 drawn to scale and aligned by their central PDZ domain. Both proteins contain two N-terminal domains (PDZ1+PDZ2 for PSD-95 and two L27 domains for MPP2) in addition to the carboxyl-terminal “MAGUK core” domains PDZ-SH3-GK. Note the differences in the length of the “linker” between PDZ and SH3 domain and of the “hook” between SH3 and GK domains. **(b)** Schematic representation of the quantitative LC–MS/MS experiment using ^16^O/^18^O-labelling to identify differential interactors from adult rat brain crude synaptosomal preparations by GST pull-down of bacterially expressed GST-MPP2-SH3GK or GST-PSD-95-SH3GK. **(c)** GST pull-downs were performed in duplicates with inverted labelling and 188 interacting proteins were identified and quantified by mass spectrometry passing our threshold settings. PSD-95/MPP2 protein ratios from both replicates A and B (normalised by the ratio of GST) are plotted against each other. Proteins in the first quadrant indicate preferential enrichment to PSD-95 (ratios >> 1), while proteins in the third quadrant indicate preferential enrichment to MPP2 (ratios << 1). Proteins with ratios of approximately 1 show no preferential binding and thus reflect equal binding to both baits or background proteins that were not fully removed by the washing steps. Selected novel potential interaction partners were validated by co-immunoprecipitation (yellow, see also S6 Fig). The most significantly enriched proteins to the GST-SH3GK construct of MPP2 (green cluster) are 7 different GABA_A_ receptor subunits, of which α1, α2, and β3 have been validated for direct interaction with MPP2 (see also Fig 4). For further details, see also Table 1 and S1 Data. GABA, γ-aminobutyric acid; LC–MS/MS, liquid chromatography with tandem mass spectrometry; MAGUK, membrane-associated guanylate kinase; MPP2, membrane protein palmitoylated 2; PSD, postsynaptic density.

The observed peripheral synaptic localisation of MPP2 relates to that of its PDZ ligand binding partner SynCAM 1 [13,16]. PSD-95 is located in central subsynaptic nanodomains that likewise correlate with the localisation of its PDZ domain-binding partners, i.e., glutamate receptors and auxiliary proteins [18–21]. The SH3 and GK domains, which are typically located at the carboxyl terminus of MAGUK scaffold proteins (for overview of MPP2 and PSD-95 domain architecture, see Fig 3A), also participate in scaffold complex formation. Importantly, it has been shown that the GK domain of MAGUK proteins is an inactive guanylate kinase [22] that has evolved into an important protein interaction domain. An intramolecular interaction between the SH3 and GK domains of MAGUKs has been well characterised, and several studies also support the idea that these domains of the MAGUK protein PSD-95 are involved in regulated multiprotein complex formation [23,24]. While numerous binding partners for this region of PSD-95 have been described, interactors specific for the SH3GK domain of MPP2 have not been identified so far.

Given the observed peripheral localisation of MPP2, we hypothesised that identification of its carboxyl-terminal interaction partners might illuminate protein complexes that differ from those organised by the central PSD-95 scaffold molecule. To explore this idea, we performed a comparative and quantitative proteomics approach (see Fig 3B for experimental design). Using bacterially expressed GST-MPP2-SH3GK and GST-PSD-95-SH3GK, we pulled down SH3GK-binding proteins from adult rat crude synaptosome preparations. Interacting proteins were eluted from the beads and separated by SDS-PAGE. Enzymatic ^16^O/^18^O-labelling was used for relative quantification of proteins by nanoLC–MS/MS analysis. In replicate A, proteins enriched by MPP2-SH3GK carried the naturally highly abundant ^16^O isotope, while proteins enriched by PSD-95-SH3GK were labelled by ^18^O using H_2_^18^O during tryptic in-gel digestion (see Fig 3B). In replicate B, labels were switched. In total, we reproducibly identified and quantified 188 proteins (see S1 Data). Plotting the protein heavy/light ratios (H/L) from replicate A (Fig 3C, X-axis) against the light/heavy ratios (L/H) from replicate B (Fig 3C, Y-axis) shows proteins enriched to PSD-95-SH3GK in the first quadrant and proteins enriched to MPP2-SH3GK in the third quadrant (Fig 3C). Background proteins (not completely washed from the beads) and proteins that bind to both SH3GK constructs show ratios of about 1. From all identified proteins, 83% have been previously reported in human and/or mouse PSD preparations [25], confirming the validity of our approach for identifying true PSD proteins. Further evidence illustrating the strength of our strategy is the fact that several known interactors of PSD-95 (including, e.g., Map1a, MeCP2, CaMKII, and Fxr1) were identified among proteins enriched in the GST-PSD-95-SH3GK pull-down (Fig 3C). Moreover, the absence of typical interaction partners of the PSD-95 and MPP2 N-terminal domains, such as N-methyl-D-aspartate receptors (NMDARs) and SynCAM 1, emphasises the specificity of our approach.

Importantly, proteins found consistently in the MPP2 pull-downs reflect putative novel synaptic MPP2 binding proteins. Of the newly identified proteins present in the GST-MPP2-SH3GK pull-down samples (see S1 Data), most have been found in PSD preparations before [25], which is in line with our previous work highlighting MPP2 as an important postsynaptic scaffold.

Several putative novel MPP2 interaction partners were selected for validation and further study (see Fig 3C, highlighted proteins; see also Table 1 for more details). We demonstrated that both Gnao1 and Arhgef2, proteins involved in signalling cascades relevant for postsynaptic function [26,27], indeed interact with MPP2 in co-immunoprecipitation assays (see S6 Fig). We also validated a clear interaction between MPP2 and the membrane-associated synaptic proteins Farp1 and Pip5k1c (S6 Fig).

**Table 1:** Validated novel interaction partners for MPP2 (for co-immunoprecipitation data, see Fig 4 and S6 Fig).

Name	UniProt accession ID	Description	Remarks
GABA_A_R β3	GBRB3_RAT	Gamma-aminobutyric acid receptor subunit beta-3	Representative brain expressed GABA_A_ receptor subunit from the β family
GABA_A_R α1	GBRA1_RAT	Gamma-aminobutyric acid receptor subunit alpha-1	Predominant GABA_A_ receptor subunit in the brain
GABA_A_R α2	GBRA2_RAT	Gamma-aminobutyric acid receptor subunit alpha-2	Common GABA_A_ receptor subunit in multiple brain tissues
Pip5k1c	PI51C_RAT	Phosphatidylinositol 4-phosphate 5-kinase type-1 gamma	Binds to FERM domains, activated by Rho/Rho GEF signalling
Farp1	FARP1_RAT	FERM, ARHGEF, and pleckstrin domain-containing protein 1	SynCAM interactor
Ppp3ca	PP2BA_RAT	Serine/threonine-protein phosphatase 2B catalytic subunit alpha isoform	Calcineurin subunit; known GABA_A_ R interactor
Arhgef2	ARHG2_RAT	Rho guanine nucleotide exchange factor 2	Interacts with AMPA receptors
Gnao1	GNAO_RAT	Guanine nucleotide-binding protein G(o) subunit alpha	Transducer in transmembrane signalling systems

Importantly, in addition to revealing previously unknown binding partners for MPP2, our comparative quantitative MS strategy provided us with important information on how the MPP2 interactome relates to that of PSD-95. Of particular interest, the set of proteins most significantly enriched in the GST-MPP2-SH3GK pull-down comprised multiple GABA_A_ receptor subunits (highlighted in green in Fig 3C; see also Table 1 and S1 Data). Seven different GABA_A_ receptor subunits, namely α1, α2, α4, β1, β2, β3, and δ, were enriched more than 7-fold. In combination, these subunits are able to form complete heteropentameric receptors [28,29], illustrating the potential for MPP2 molecules to interact at multiple sites with a fully functional GABA_A_ receptor. These results are striking, as GABA_A_ receptors are not known to be expressed at high levels at the PSD of glutamatergic synapses where MPP2 is enriched.

In this context, it is interesting that MPP2 also interacts with the calcium-dependent, calmodulin-stimulated protein phosphatase (Calcineurin) subunit Ppp3ca (see Fig 3C and Table 1 and S6 Fig), which is known to influence GABA_A_ receptor signalling [30]. Together, these data support the novel idea that MPP2 could be involved in GABA_A_ receptor-mediated processes at the PSD of glutamatergic synapses.

### GABA_A_ receptor subunits are novel synaptic interaction partners of MPP2

**Fig 4:**
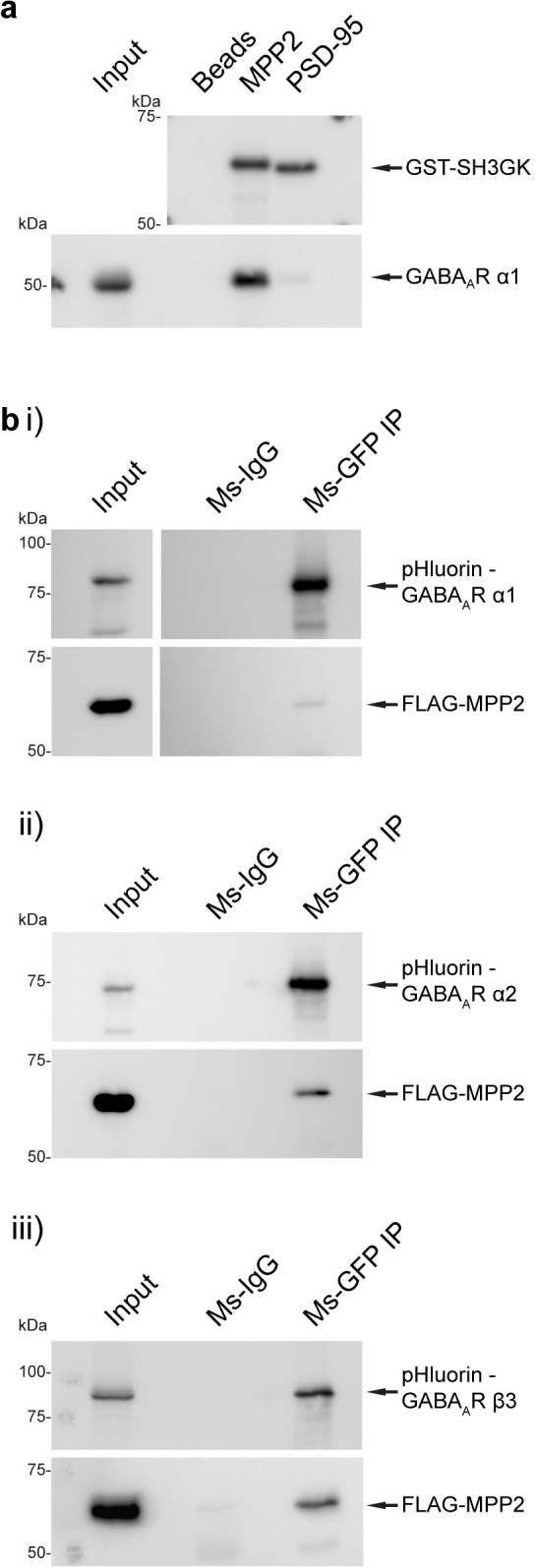
Validation of GABA_A_ receptor subunits as novel interaction partners of MPP2. **(a)** Bacterially expressed GST-MPP2-SH3GK and GST-PSD-95-SH3GK were incubated with crude brain synaptosome preparations. After GST pull-down, compared to bead control, GABA_A_R α1 was efficiently enriched in the GST-MPP2-SH3GK pull-down, as detected by western blot with αGABA_A_R α1 and αGST antibodies. **(b)** GABA_A_ receptor subunit constructs with an N-terminal pHluorin were acquired from Addgene and expressed in CHL V79 cells together with N-terminally FLAG-tagged MPP2. Pull-down of (i) GABA_A_R α1, (ii) GABA_A_R α2, and (iii) GABA_A_R β3 with Ms αGFP or normal Ms-IgG resulted in co-precipitation of FLAG-MPP2 as detected by western blot with αFLAG-HRP and αGFP antibodies. GABA, γ-aminobutyric acid; MPP2, membrane protein palmitoylated 2; PSD, postsynaptic density.

In subsequent experiments, we focused on the finding that the proteins most significantly enriched in the MPP2-SH3GK pull-down were multiple GABA_A_ receptor subunits. Importantly, our comparative pull-down experiments using GST-tagged SH3GK domains of MPP2 and PSD-95 validate our mass spectrometry data (Fig 3) and demonstrate that MPP2—in contrast to PSD-95—binds effectively to the representative subunit GABA_A_R α1: While the MPP2 SH3GK domain efficiently pulls out the endogenous GABA_A_R α1 from crude synaptosome preparations, the PSD-95 SH3-GK domain does not (Fig 4A).

To further investigate this unexpected result, we overexpressed several of these GABA_A_ receptor subunits together with full-length FLAG-MPP2. Upon pull-down of the full-length pHluorin-tagged receptor subunits with Ms-αGFP antibody, we could detect MPP2 in the precipitate (Fig 4Bi), suggesting that GABA_A_R α1 is indeed a new binding partner for MPP2. Using the same strategy, we could also confirm a direct interaction between MPP2 and the GABA_A_R α2 (Fig 4Bii) and GABA_A_R β3 (Fig 4Biii) subunits, further validating our MS results, and providing support for the idea that the MPP2-GABA_A_ receptor interaction is real and important.

### Modulating MPP2 expression affects inhibitory synaptic transmission

**Fig 5:**
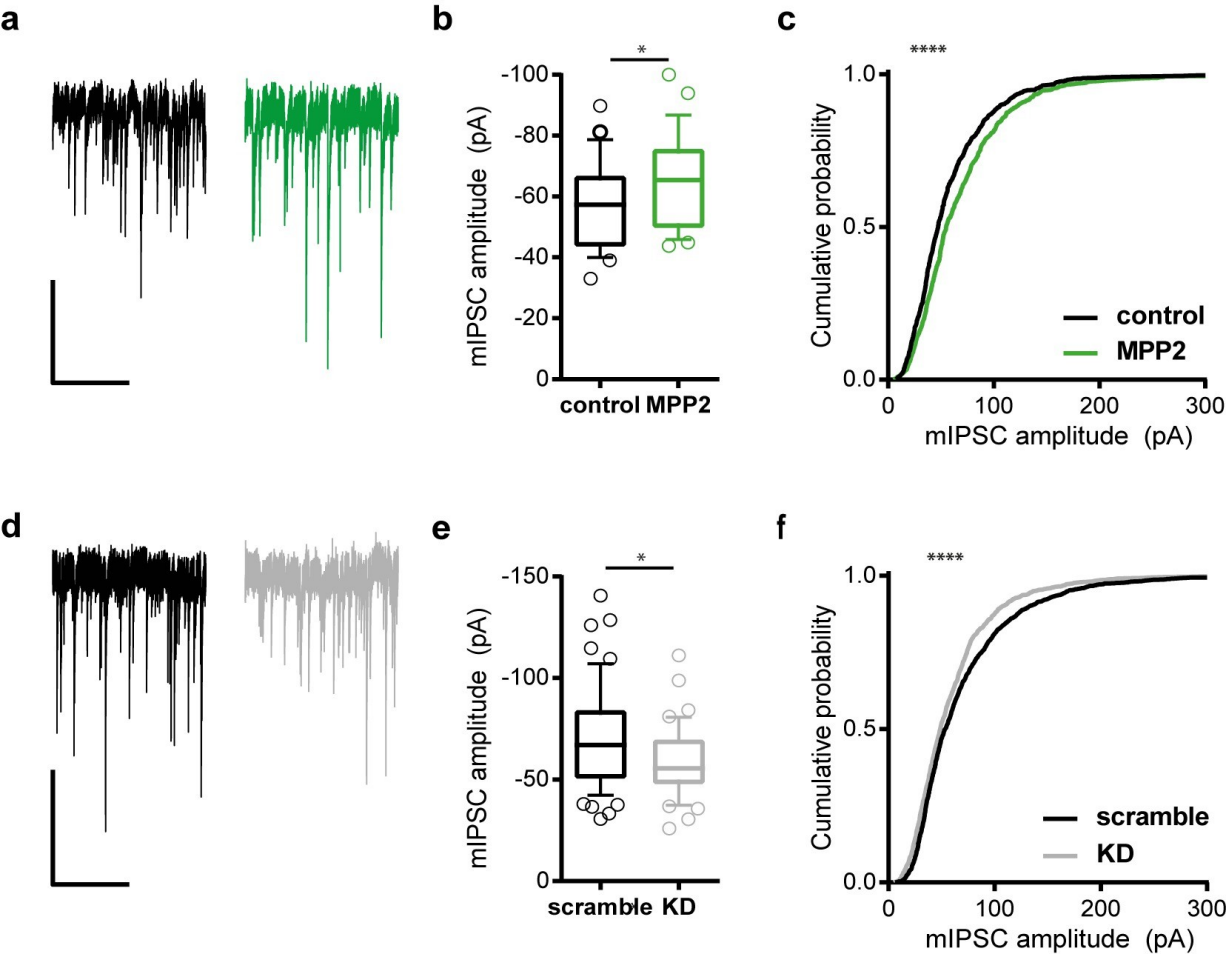
MPP2 expression levels influence mIPSC amplitudes. **(a)** Example traces of mIPSC in untreated (black) and MPP2-overexpressing (green) neurons. Scale bar: 100 pA/5s. **(b)** Overexpression of MPP2 leads to an increase in average mIPSC amplitude (unpaired *t* test *p* = 0.0403). Error bars of the boxplots correspond to the 10th and 90th percentile; control: *n* = 27; overexpression: *n* = 27. **(c)** Cumulative distribution of individual mIPSC amplitudes show a right-shift (larger amplitudes) in MPP2 overexpressing neurons. Kolmogorov–Smirnov test *p* < 0.0001. **(d)** Example traces of mIPSC from neurons transduced with control shRNA (black) and MPP2 knockdown shRNA (grey). Scale bar: 100 pA/5s. **(e)** Knockdown of MPP2 leads to a decrease in average mIPSC amplitude (Mann–Whitney test *p* = 0.0277). Error bars of the boxplots correspond to the 10th and 90th percentile; scramble: *n* = 58; KD: *n* = 40. **(f)** Cumulative distribution of individual mIPSC amplitudes shows a left-shift (smaller amplitudes) in MPP2 knockdown neurons (Kolmogorov–Smirnov test *p* < 0.0001). Underlying data can be found in S2 Data. mIPSC, miniature inhibitory postsynaptic current; MPP2, membrane protein palmitoylated 2.

In order to explore the idea that MPP2 might indeed act as a scaffold for GABA_A_ receptors and thereby modulate their function, we recorded mIPSCs in our dissociated hippocampal neuron cultures (Fig 5A), which generally reflect synaptic GABA_A_R-mediated responses to GABA release. Overexpression of MPP2 resulted in an increase in the average recorded mIPSC amplitude, when compared with that recorded in uninfected control neurons (Fig 5B), while the average frequency of events was unchanged by overexpression of MPP2. Cumulative frequency distribution of a fixed number of events per cell (Fig 5C) highlighted the significance of the difference between MPP2-overexpressing and control neurons, with regard to mIPSC amplitude (Kolmogorov–Smirnov test *p* < 0.0001), whereas cumulative distribution of individual interevent intervals (IEIs) shows no difference between control and MPP2-overexpressing neurons (Kolmogorov–Smirnov test *p* = 0.1227). ShRNA-mediated knockdown of MPP2 correspondingly reduced mIPSC amplitudes when compared to control neurons transduced with scrambled shRNA (Fig 5D), in line with a postsynaptic function of MPP2. The frequency of mIPSCs differs slightly between MPP2 knockdown and control neurons, observable only in the cumulative distribution of individual IEIs, which indicates a right-shift (longer IEIs) in MPP2 KD neurons (Kolmogorov–Smirnov test *p* = 0.0004). This difference in frequency may reflect a secondary effect, i.e., a detection failure resulting from the reduced amplitudes of recorded events. MPP2 knockdown shRNAs are specific for MPP2 among the MPP family of MAGUKs, and efficient knockdown of endogenous MPP2 was confirmed (see control western blots in S7 Fig), suggesting that these observed changes in mIPSC amplitude indeed result from a reduced MPP2 expression along the dendrites and spines of glutamatergic neurons, where we know endogenous MPP2 is enriched.

### MPP2 is not part of classical Gephyrin-positive inhibitory postsynaptic scaffolds but colocalises with endogenous GABA_A_R subunits in a subpopulation of dendritic spines

**Fig 6:**
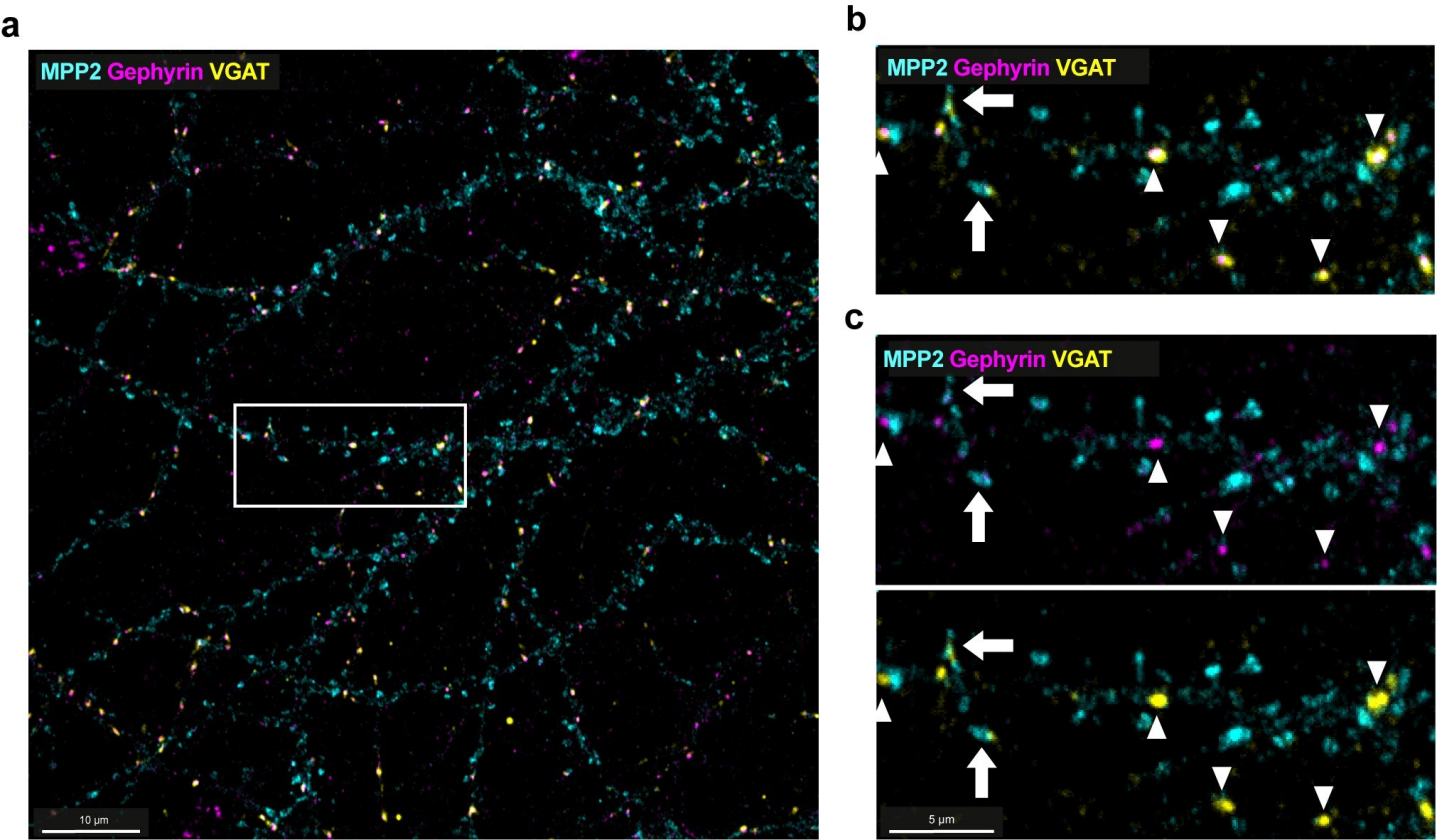
MPP2 is not expressed at classical inhibitory synapses. **(a)** Confocal imaging maximum projection of primary rat hippocampal neurons fixed at DIV21 and stained for endogenous proteins MPP2 (cyan) together with the inhibitory postsynaptic scaffold protein Gephyrin (magenta) and the inhibitory presynaptic marker VGAT (yellow). White box indicates region of detail view in b and c. Scale bar: 10 μm. **(b, c)** Detail view of region indicated in a. MPP2 is clearly enriched in dendritic spines along neuronal dendrites (which have lower MPP2 expression) and generally distinct from inhibitory synapses (arrowheads) as marked by Gephyrin staining with directly adjacent VGAT. MPP2 and Gephyrin staining does not significantly overlap, but occasionally MPP2-positive spines have adjacent VGAT puncta (arrows). Scale bar: 5 μm. DIV, days in vitro; MPP2, membrane protein palmitoylated 2.

Our in vitro biochemical experiments clearly show that multiple GABA_A_ receptor subunits strongly interact with MPP2, and physiological recordings suggest that there is indeed a functional interaction between MPP2 and GABA_A_ receptors. In light of the fact that GABA_A_ receptors are best known for their importance at inhibitory synapses where Gephyrin is the predominant scaffold (for review, see [31]), this result was surprising. To further assess the subcellular localisation of this functional interaction, we immunostained dissociated rat primary hippocampal neurons for the relevant endogenous proteins (Fig 6). Neuronal cultures were fixed at days in vitro (DIV)21 and first stained for MPP2 (cyan) and Gephyrin (magenta), together with VGAT (yellow) as a marker for inhibitory presynapses. As expected, Gephyrin staining highlighted puncta along the dendrites that were typically associated with adjacent presynapses positive for the presynaptic marker VGAT (see arrowheads in Fig 6B and 6C). These Gephyrin-positive puncta along the dendrites were generally distinct from MPP2-positive spines (see Fig 6A overview image; see also inset in Fig 6C top), suggesting that the observed functional and biochemical interaction between MPP2 and GABA_A_ receptors is not occurring routinely at classical Gephyrin-positive inhibitory postsynapses. We do observe some dendritic spines that clearly express MPP2 with an adjacent VGAT signal (marked with arrows, see Fig 6C bottom), highlighting that a subset of MPP2-enriched spines may indeed receive GABAergic inputs.

**Fig 7:**
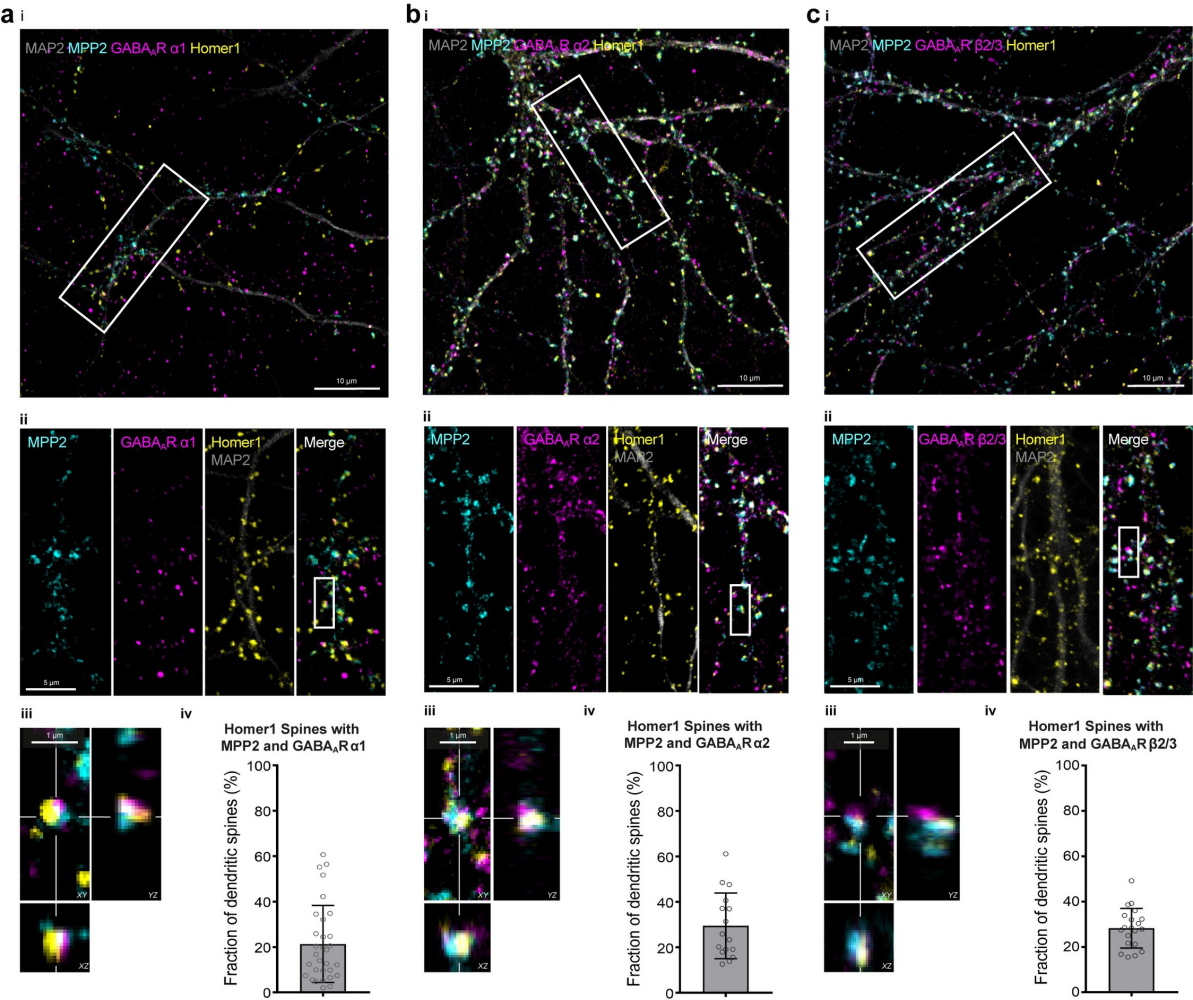
GABA_A_R subunits colocalise with MPP2 in a subset of dendritic spines. Primary E18 rat hippocampal neurons were fixed at DIV21 and immunostained for the endogenous proteins MAP2 (microtubule-associated protein 2, grey), MPP2 (cyan), and Homer1 (yellow) together with either **(a)** GABA_A_R α1, **(b)** GABA_A_R α2, or **(c)** GABA_A_R β2/3 (all magenta) using respective primary and Alexa fluorophore-coupled secondary antibodies, and visualised by confocal microscopy. (i) Maximum projected overview of primary to secondary dendrite branches. Box indicates location of detail image in ii. Scale bar: 10 μm. (ii) Maximum projection composite of 4-colour confocal immunofluorescence image of a primary to secondary dendrite branch point of a mature (DIV21) hippocampal neuron. MPP2 is located in the majority of dendritic spines marked with Homer1, while GABA_A_R subunits colocalise with a subset of these spines. Additionally, solely GABA_A_R α1-, α2- or β2/3- positive puncta likely represent inhibitory synapses at the dendrite. Box indicates location of detail image in iii. Scale bar: 5 μm. (iii) Enlarged single plane image with corresponding orthogonal views of a dendritic spine exhibiting immunofluorescence staining for Homer1, MPP2, and GABA_A_R α1, α2 or β2/3, respectively. Scale bar: 1 μm. (iv) Quantification of the fraction of excitatory synapses marked by Homer1, which also show MPP2 expression together with GABA_A_R α1, α2, or β2/3 immunofluorescence. Please see Materials and methods for details. (mean ± SD; *n* = 16–31 images from *N* = 3 to 4 independent experiments). Underlying data can be found in S2 Data. DIV, days in vitro; GABA, γ-aminobutyric acid; MPP2, membrane protein palmitoylated 2.

We next stained for GABA_A_R α1 and MPP2 together with the dendritic marker MAP2 (microtubule-associated protein 2) and the PSD marker Homer1, with respective primary and secondary antibodies. MPP2 (Fig 7, cyan) is present in almost all dendritic spines positive for the PSD marker Homer1 (Fig 7, yellow). Upon analysis of secondary dendrite segments (Fig 7Aii), we observed GABA_A_R α1 signals (magenta) not only interspersed along MAP2-positive dendritic branches (grey), where expected, but also colocalising directly with MPP2 and Homer1 in a subset of dendritic spines (Fig 7Aiii). We developed an automated segmentation pipeline to select Homer1-positive dendritic spines, and we then tested those spines for the presence of MPP2 and GABA_A_R α1 signals within a typical spine head diameter around the centre of the segment. Using this approach, we were able to assess the prevalence of the co-occurrence of these 3 markers within a single spine (as shown in Fig 7Aiii and 7Aiv; for details, see Materials and methods). By analysis of our fixed primary hippocampal neurons at or close to primary to secondary dendrite branch points, we found that approximately 20% of all Homer1-positive spines also express MPP2 and GABA_A_R α1 (Fig 7Aiv; mean ± SD; median = 16.7%).

We also stained fixed neurons for the GABA_A_R α2 (Fig 7B) and the beta subunits GABA_A_R β2/3 (Fig 7C) and likewise observed overlapping expression with MPP2 and Homer1 in a subset of synapses.

**Fig 8:**
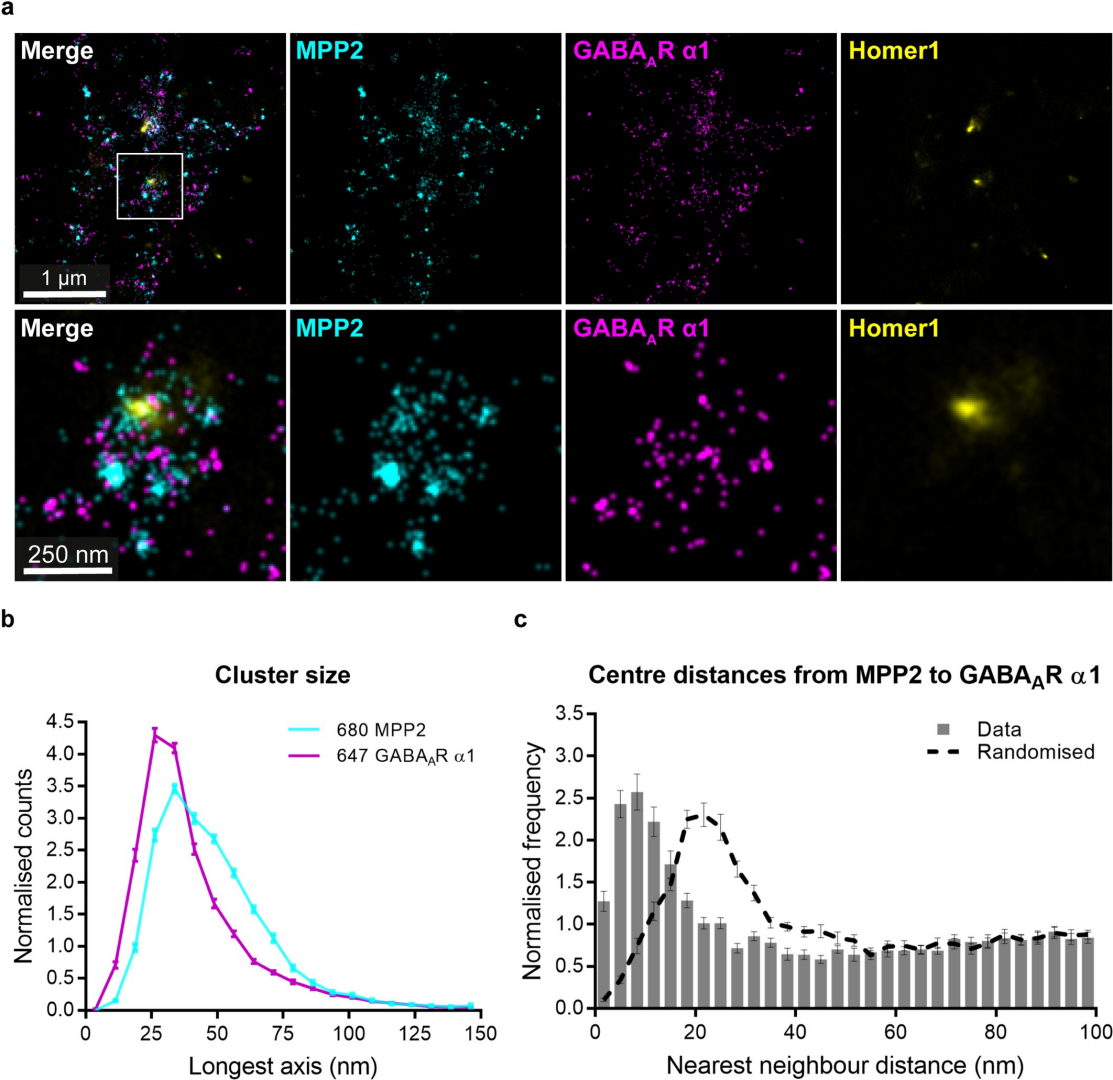
MPP2 and GABA_A_R α1 form highly overlapping nanoclusters. Mature (DIV21) primary rat hippocampal neurons were immunostained for endogenous MPP2 (cyan, second column), GABA_A_R α1 (magenta, third column), and the PSD marker Homer1 (yellow, fourth column) and subjected to triple-colour *d*STORM imaging. **(a)** Overview of a dendritic segment (upper row). White box indicates location of detail image below. Zooming onto an individual synapse expressing Homer1, MPP2, and GABA_A_R α1 (lower row) reveals a close association between clusters of MPP2 and GABA_A_R α1 and the bracelet-like arrangements of MPP2 surrounding a centrally positioned Homer1 cluster. Scale bars: overview 1 μm; detail 250 nm. **(b)** Histograms illustrating the distribution of nano-cluster sizes (longest axis) for MPP2 (cyan) and GABA_A_R α1 (magenta). Clusters were detected via DBSCAN. Mean ± SEM; *n* = 26 images from *N* = 3 independent experiments. **(c)** NN analysis of MPP2 and GABA_A_R α1 protein clusters after DBSCAN. NN distances were calculated from the cluster centres. Closest GABA_A_R α1 to MPP2 were analysed (grey bars). Dashed lines represent the random control by toroidal shift. Note the close association of both clusters (approximately 10 nm distance between both centres), which is well below the cluster sizes (20 to 40 and 30 to 60 nm, see b) and shifted after randomisation. Mean ± SEM; *n* = 26 images from *N* = 3 independent experiments. Please see S8 Fig for related analysis in the reverse direction. Underlying data can be found in S2 Data. DIV, days in vitro; *d*STORM, direct stochastic optical reconstruction microscopy; GABA, γ-aminobutyric acid; MPP2, membrane protein palmitoylated 2; NN, nearest neighbour; PSD, postsynaptic density.

In light of the close association of MPP2 with the cell adhesion molecule SynCAM 1 at the PSD periphery, we took advantage of superresolution microscopy to explore the subcellular localisation of the MPP2-GABA_A_ receptor association. We applied 3-colour *d*STORM imaging to DIV21 primary hippocampal neurons, stained for endogenous MPP2, the representative subunit GABA_A_R α1, and Homer1 (as a PSD marker). Specifically, we performed *d*STORM experiments of the 3 proteins at Homer1-positive PSDs, taking advantage of spectral de-mixing (SD) *d*STORM in combination with a successively recorded “conventional” *d*STORM experiment for the third channel. While Homer1 (CF 568) was recorded in “conventional” *d*STORM mode and required bead-based channel alignment, MPP2 (CF 680) and SynCAM 1 (Alexa 647) were imaged in spectral de-mixing mode, which is entirely free from registration errors. Using this strategy, we were able to observe clusters of GABA_A_R α1 (Fig 8A, magenta) along the dendrite, as well as MPP2 clusters (Fig 8, cyan) that are associated with Homer1-positive PSDs (Fig 8, yellow).

Upon closer examination of individual synapses, the bracelet-like arrangement of MPP2 clusters surrounding the PSD is apparent. Most strikingly, we observe small clusters of GABA_A_R α1 that are tightly associated with MPP2 (Fig 8A, lower panel). Detailed analysis of the MPP2 and GABA_A_R α1 clusters provides further evidence for the close association of these 2 proteins: MPP2 clusters are 30 to 60 nm in diameter (Fig 8B, cyan), while GABA_A_R α1 clusters are somewhat smaller (20 to 40 nm in diameter; see Fig 6B, magenta). The NN analysis between MPP2 and GABA_A_R α1 clusters (centre-to-centre distances) peaks at approximately 10 nm (Fig 8C), which is well below the observed cluster sizes and also beyond the resolution of our *d*STORM system. Thus, we conclude that both proteins are highly overlapping at the PSD periphery.

Together with our in vitro and physiological data, our confocal and *d*STORM imaging results suggest that MPP2 and GABA_A_ receptors functionally interact in a subpopulation of dendritic spines and that MPP2 acts as a scaffold protein potentially involved in the modulation of GABA_A_ receptor function at the PSD periphery.

## Discussion

We are interested in how subsynaptic nanodomains at glutamatergic synapses are organised such that they can orchestrate synaptic function, which is highly dynamic. Here, we show that PSD-95 and MPP2, 2 related synaptic MAGUKs, are close, but distinctly localised at the PSD of glutamatergic synapses. Using superresolution imaging strategies, we observe that SynCAM 1 and MPP2 are located in juxtapose association towards the outside of the PSD, with both proteins surrounding central PSD-95 protein complexes at radial distances that reflect a peripheral PSD localisation, given typical expected PSD sizes [17]. These observations, combined with the fact that MPP2 interacts directly with the peripheral SynCAM 1 (but not the more central AMPAR-auxiliary subunits, TARPs), led us to pursue the idea that MPP2 may act as a scaffold for protein complexes that are distinct from those at the core of the PSD. Using a comparative and quantitative proteomics approach, we demonstrate here that the SH3GK domains of MPP2 and PSD-95, which are structurally similar, indeed interact with distinct sets of cytosolic and membrane proteins present within dendritic spines. Importantly, we identified several novel MPP2-interacting proteins, including multiple GABA_A_ receptor subunits as well as signalling molecules with established roles in the regulation of inhibitory transmission. Modulating MPP2 expression levels in hippocampal neuronal cultures resulted in small but significant changes in the average amplitude of recorded mIPSCs, in line with a functional interaction between MPP2 and a specific subset of GABA_A_ receptors. The analysis of cumulative probability of the individual IPSC amplitudes shows that, in particular, the larger event amplitudes were affected by modulating the expression of MPP2, suggesting that it is the stronger GABAergic synapses that are more likely to be affected. Determining whether these synapses of interest are those innervated by a specific interneuron subtype, and if they can be further characterised with regard to synapse composition and dendritic localisation, remains to be determined. Detailed imaging suggests that indeed the relevant pool of inhibitory postsynapses is unique. Analysis of endogenous MPP2 and multiple GABA_A_ receptor subunits highlighted a subset of dendritic spines that clearly express both GABA_A_ receptors and MPP2, which are positioned in tight association at the PSD periphery, and MPP2 was not generally expressed at Gephyrin-positive inhibitory postsynapses at the soma and throughout the dendrites. Together, these data indicate a role for MPP2 as a scaffold protein that coordinates functional subsynaptic nanodomains that are distinct from those defined by PSD-95 and highlight its role as a potentially important mediator of inhibitory signalling in direct association with glutamatergic synapses.

Our combined imaging strategies illustrate that MPP2 and SynCAM 1 sit directly next to each other towards the outside of the PSD, positioned optimally to regulate the formation of peripheral subsynaptic nanodomains, and our comparative quantitative proteomics approach provides further information on the nature of these protein complexes. Importantly, well-known PSD-95-interacting proteins, including, e.g., Map1a and MeCP2, were highly enriched in the PSD-95-SH3GK pull-downs, whereas Farp1, a well-characterised SynCAM 1 interactor and modulator of SynCAM-mediated processes [32], was found to be significantly enriched in our MPP2-SH3GK pull-downs. These data in particular illustrate the utility of our strategy for identifying unknown MPP2 interactors of potential importance. Among the crude synaptosome proteins present in the MPP2-SH3GK pull-down, we consistently identified seven GABA_A_ receptor subunits, i.e., more than one-third of all known subunits [33]. The fact that so many GABA_A_ receptor subunits were in the top hits among MPP2-associated proteins supports the idea that the MAGUK-GABA_A_ receptor association is MPP-specific and that these receptors do not generally bind with high affinity to the SH3GK domains of other synaptic MAGUKs. In co-immunoprecipitation experiments, we could confirm a direct interaction of MPP2 with the GABA_A_ receptor subunits GABA_A_R α1, α2, and β2/3, and in our primary hippocampal neuron cultures, we detect these endogenous GABA_A_ receptor subunits at a subset of Homer1-positive glutamatergic synapses that express MPP2. We were unable to demonstrate a biochemical interaction of the endogenous proteins in pulldown experiments with available antibodies, likely due to the fact that most dendritic GABA_A_ receptors do not reside in spines bound to MPP2 but instead interact tightly with the classical inhibitory scaffold protein Gephyrin, which is abundant at dendritic and somatic inhibitory postsynapses. Nonetheless, our physiological data, highlighting that changes in MPP2 expression can indeed have functional consequences on measures of inhibitory transmission combined with the fact that we do not observe significant MPP2 expression across Gephyrin-positive inhibitory postsynapses throughout our neurons, provide support for the idea that there is a functional role for MPP2-GABA_A_ receptor protein complexes at specific inhibitory synapses at dendritic spines.

GABA_A_ receptors mediate inhibitory transmission onto dendrites at diverse locations of the dendritic arbour (for reviews, see [30,31,34,35]). Similar to the subsynaptic nanodomains formed by receptors and scaffold proteins at glutamatergic synapses [5,6], superresolution imaging techniques have revealed that GABA_A_ receptors and their associated Gephyrin protein scaffolds form analogue subsynaptic compartmentalisations that are regulated dynamically in response to synaptic activity [36–38]. Electron microscopy studies have revealed that GABA_A_ receptor complexes are present not only on dendritic shafts but also in dendritic spines and in close proximity to PSDs of glutamatergic synapses in cortical neurons [39]. This perisynaptic localisation of GABA_A_ receptor subunits has also been monitored by other groups in a developmental context [40]: These authors demonstrated expression of GABA_A_R subunits (specifically α4 and δ, both of which we identified in our MS experiments) at dendritic spines, with onset of expression during maturation. A developmentally regulated expression of GABA_A_ receptors at spines is in line with a role for establishment of an MPP2-GABA_A_R interaction in response to glutamatergic activity at maturing synapses.

The importance of GABA_A_ receptors within dendritic spines and their role in regulating Ca^2+^ influx and general excitatory signal transmission [41] has become a topic of considerable interest (for reviews, see [34,35]). In a recent study, the authors observed that approximately 10% of cortical dendritic spines harbour inhibitory synapses, and quantitative comparative analysis of doubly- versus singly-innervated spines illuminated the unique properties of this subgroup of spines [42]. Other studies highlight that GABA_A_ receptor mobility between inhibitory synapses is an important mechanism by which inhibitory transmission is regulated and that this process can be modulated by activation of ionotropic glutamate receptors and subsequent trapping of desensitised GABA_A_ receptors at glutamatergic synapses [43]. There are also several studies highlighting links between NMDAR activation and plasticity at inhibitory synapses [44–48].

Clearly, there are multiple functional links between GABA_A_ receptor signals and glutamatergic transmission at the PSD, and the idea that a subset of dendritic spines might have unique functional properties that depend on the type of GABA_A_-associated scaffolds within them is supported by the literature [34, 42]. However, the physical interactions that enable such functional connections have not been elucidated to date. Our imaging studies highlight that MPP2 is positioned optimally—next to the core components of glutamate receptor signalling complexes, but at the periphery of the PSD and in close association with GABA_A_ receptors in dendritic spines—to play a structural role in orchestrating the complex formation that would be required to enable dynamic links between glutamatergic transmission and GABA_A_ receptor signalling.

**Fig 9:**
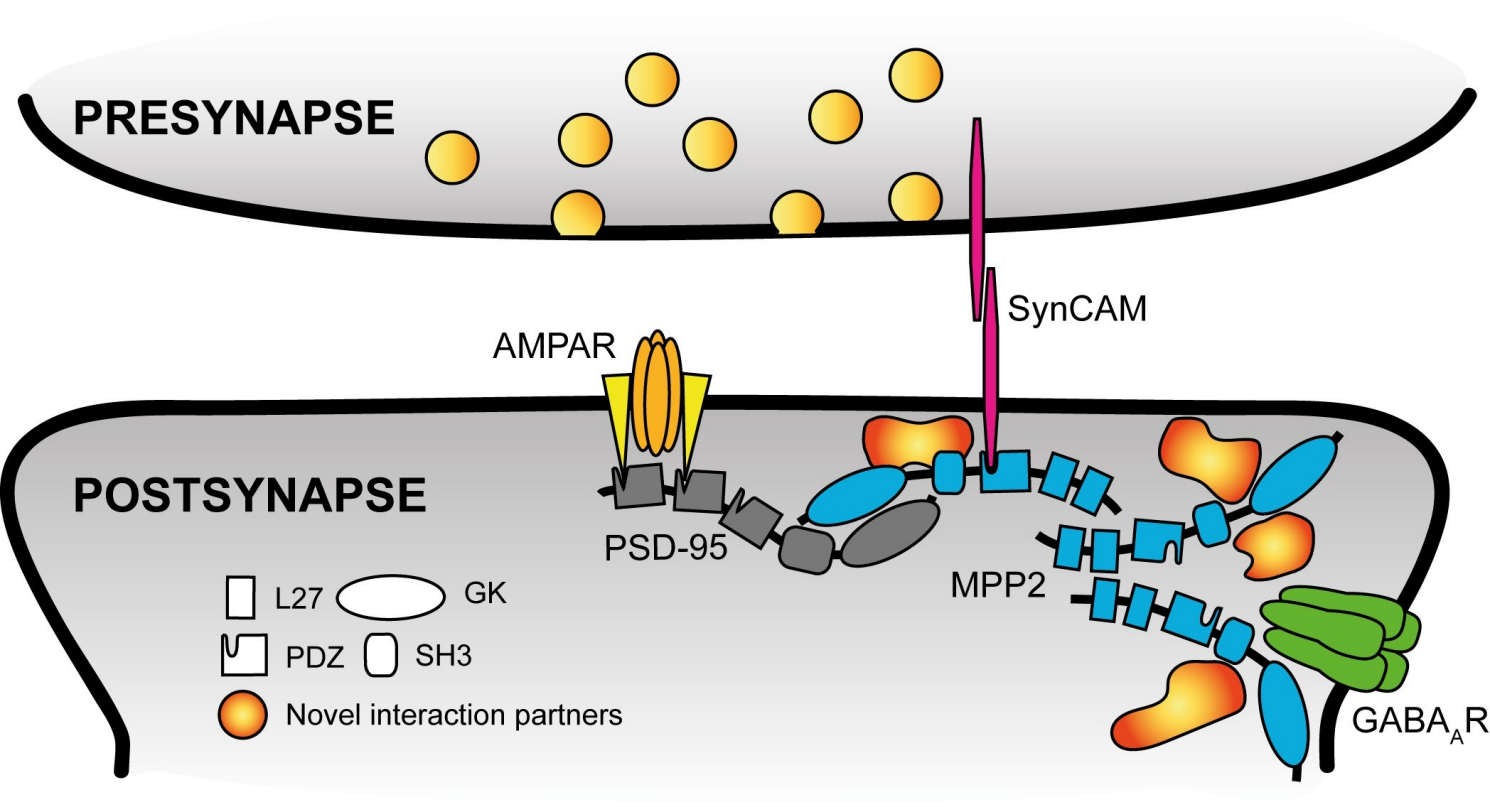
Schematic summary of novel MPP2 interactors. Our data indicate an important role of MPP2 (blue) in the subsynaptic compartmentalisation of dendritic spines by connecting central components of AMPA receptor (orange) complexes, like TARPs (yellow) and PSD-95 (grey) not only to cell adhesion proteins like SynCAM 1 (magenta) and other scaffold and regulatory proteins (like the novel interaction partners PIP5k1c or Ppp3ca), but most importantly inhibitory GABA_A_ receptors (green). For MAGUK proteins, the individual domain structure is indicated. GABA, γ-aminobutyric acid; MAGUK, membrane-associated guanylate kinase; MPP2, membrane protein palmitoylated 2; PSD, postsynaptic density.

Our data, which demonstrate a functional link between GABA_A_ receptors and MPP2 and in parallel highlight their overlapping expression at the periphery of glutamatergic synapses, lead us to propose a model in which MPP2 multimolecular complexes serve as adaptors that potentially enable a bidirectional crosstalk between GABA_A_ receptor-mediated inhibitory regulation and glutamatergic transmission in dendritic spines (see Fig 9). Our model is further supported by the fact that one of the other novel MPP2 interaction partners identified in this study has previously been associated with inhibitory signalling through GABA_A_ receptors: the calcium- and calmodulin-dependent serine/threonine protein phosphatase (Calcineurin) subunit Ppp3ca directly interacts with GABA_A_ receptors [44,49,50] and modulates the effects of NMDAR-mediated signalling on inhibitory synaptic plasticity [45,51].

In order to achieve coordinated crosstalk between glutamatergic synapses and neighbouring inhibitory receptors, a local structural compartmentalisation is essential. Our study provides insights into the physical interactions that coordinate such compartmentalisation in dendritic spines. We demonstrate that the MPP2 scaffold protein serves to link core proteins of glutamatergic synapses with GABA_A_ receptors and associated signalling molecules in dendritic spines and thereby illuminate its potential to facilitate dynamics between excitatory and inhibitory transmission at the PSD of glutamatergic synapses. Future investigations into the precise nature of such MPP2-mediated crosstalk will contribute to our understanding of excitation–inhibition balance, which is highly relevant for circuit function in healthy and diseased states.

## Materials and methods

### Primary neuronal cultures

Primary hippocampal neurons were prepared as described before [13], in accordance with the Directive 2010/63/EU of the European Parliament on the protection of animals used for scientific purposes. Protocols for animal sacrifice were approved by the Regional Office for Health and Social Affairs in Berlin (“Landesamt für Gesundheit und Soziales; LaGeSo”) and the animal welfare committee of the Charité and carried out under permits T0280/10 and T-CH 0002/21. Briefly, E18 Wistar rat pups were decapitated, hippocampi isolated, and digested with Trypsin/EDTA (Lonza, Switzerland). Digest was stopped with DMEM/10% FBS (Biochrom, Germany), followed by washing in DMEM (Lonza). Tissue was then dissociated and plated at approximately 10^5^ cells per cm^2^ in neuron culture medium (Neurobasal (Lifetech, USA) supplemented with B27 (Gibco, USA) and 500 μM L-glutamine (Lonza)) onto coverslips coated with poly-D-Lysine (0.2 mg/ml, Sigma, USA) and Laminin (2 μg/ml, Sigma) and maintained in a humidified incubator (37°C, 5% CO_2_).

Modulating MPP2 expression was achieved through lentivirus-mediated gene delivery of either N-terminally EGFP-tagged MPP2 (infection at DIV10), or shRNAs targeting endogenous MPP2 (infection at DIV3). All neurons were fixed or harvested at DIV21/22.

### Immunocytochemistry/immunofluorescence

Primary rat hippocampal neurons were fixed at DIV21 with 4% PFA/PBS for 10 minutes at room temperature (RT), washed thrice for 10 minutes with PBS, followed by 45 minutes quenching at RT with 50 mM NH_4_Cl to reduce auto-fluorescence. After washing with PBS, cells were permeabilised with 0.2% Triton-X/PBS for 5 minutes and washed with PBS. For *d*STORM, microscopy cells were additionally treated with Image-IT Signal Enhancer (Thermo Fisher Scientific, USA) for 30 minutes at RT and 3 washes with PBS. Cells were then blocked for at least 1 hour at RT with blocking solution (4% BSA/PBST). Primary antibodies were diluted 1:250 in blocking solution (1:500 for confocal microscopy) and incubated over night at 4°C, followed by incubation with desired secondary antibodies at 1:1,000 dilution in blocking solution for 1 hour at RT and for *d*STORM custom-labelled secondary antibodies at 1 μg/ml (approximately 7 nM) in blocking solution for 20 minutes, followed by postfixation and quenching as above. After final washing with PBS, coverslips were mounted with Fluoromount G (SBA) or Vectashield (H1000, Vector Laboratories, USA) for confocal and 3D SIM imaging, respectively.

**Primary antibodies:** αHomer1 (guinea pig, 160 004, Synaptic Systems, Germany), αMPP2 (rabbit, ab97290, Abcam, USA), αMAP2 (guinea pig, 188 004, Synaptic Systems), αPSD-95 (mouse, 75–028, NeuroMab, USA), αvGlut1 (guinea pig, 135 304, Synaptic Systems), αGFP (chicken, ab13970, Abcam), αDDDK (chicken, ab1170, Abcam), αGABA_A_R α1 (mouse, 75–136, NeuroMab), αGABAAR α2 (mouse, 75–384, NeuroMab), αGABAAR β2/3 (mouse, 75–363, NeuroMab), αSynCAM 1 (chicken, CM004-3, MBL, Japan), αGephyrin (mouse, 147 011, Synaptic Systems), and αVGAT (guinea pig, 131 308, Synaptic Systems).

**Secondary antibodies:** αGuinea pig Alexa Fluor 405 (ab175678, Abcam), αGuinea pig DyLight405 (706-475-148, Dianova, Germany), αRabbit Alexa Fluor 488 (A-21441, Invitrogen, USA), αChicken Alexa Fluor 488 (703-545-155, Jackson ImmunoResearch), αMouse Alexa Fluor 568 (A-11031, Life Technologies, USA), αRabbit Alexa Fluor 568 (A-11036, Invitrogen), αMouse Alexa Fluor 647 (715-605-150, Jackson ImmunoResearch, USA), αMouse Alexa Fluor 647 (A21236, Invitrogen), αGuinea pig Alexa Fluor 647 (A21450, Thermo Fisher Scientific), αChicken Alexa Fluor 647 (103-605-155, Dianova), αRabbit (AffiniPure, 111-005-003, Jackson ImmunoResearch, USA), αRabbit (111-007-008, Jackson ImmunoResearch) αMouse (AffiniPure, 715-005-151, Jackson ImmunoResearch), and αGuinea pig (106-007-008, Jackson ImmunoResearch).

For custom labelled secondary antibodies used in dual- and 3-colour *d*STORM experiments, please see the respective Materials and methods below.

### Confocal microscopy

#### Image acquisition

Cells were fixed and stained as described above and imaged with a Nikon A1Rsi+ confocal laser scanning microscope run with NIS Elements scan software. Image stacks were acquired with a 60× 1.4 N.A. oil immersion objective, an additional 1.5× optovar lens (Nikon, Japan) and standard emission filters at 1.8× zoom at 1024 × 1024 px as 12 planes with 0.175-μm step size in Z. Image analysis was performed in Fiji/ImageJ [52,53]. Four-colour confocal images were maximum projected and subjected to further analysis.

#### Image analysis

Image analysis was performed with a custom written FIJI/ImageJ script available at Github: https://github.com/BettinaSchmerl/MPP2. For segmentation and skeletonisation of dendritic branches labelled with MAP2, maximum projections of confocal images were subjected to Otsu’s threshold clustering algorithm, followed by Gaussian blur, binarisation, and skeletonisation. For analysis of the fraction of Homer1 spines positives for both proteins of interest, MPP2 and GABA_A_R subunits, channels were filtered (Mean filter, 1.5 radius), punctate structures identified with histogram-based threshold procedures (Otsu’s, Yen’s, and the Moments methods), followed by Gaussian blur (sigma = 1.5) and counted with the build-in “Find Maxima” tool of FIJI/ImageJ. To ensure analysis of only synapses fully enclosed by the image stack, only puncta within a 2-μm periphery to a MAP2 dendrite skeleton were considered for further analysis. In order to select spines with immunostaining of MPP2 and GABA_A_R subunit proteins, those point selections were enlarged by 0.5 μm and checked for co-occurrence of identified puncta within that perimeter, covering an average synaptic spine area. Fractions of Homer1-positive spines per image having also MPP2 and GABA_A_R subunit signal were calculated based on the overall count of Homer1-positve spines per image.

### Dual-colour *d*STORM imaging

#### Labelling of antibodies

Secondary antibodies (goat αRabbit, 111-005-003, AffiniPure or donkey αMouse, 715-005-151, AffiniPure) were diluted 1:10 in labelling buffer (0.2 M NaHCO_3_, pH 8.3). Cy3b NHS Ester (PA63101, Life Sciences, Germany) was added to the diluted antibodies in 10-fold molar excess. The samples were incubated for 1 hour at RT. To stop the reaction, 100 mM Tris pH 8.0 was added. Zebra spin desalting columns (8989, Thermo Fisher Scientific) were equilibrated with PBS. The samples were added to the column and centrifuged at 1,000 *g* for 2 minutes. The filtrate was added to a second column and centrifuged at 1,000 *g* for 2 minutes.

#### Image acquisition

All samples were imaged using a Vutara 352 superresolution microscope (Bruker, USA) equipped with a Hamamatsu ORCA Flash4.0 sCMOS camera for superresolution imaging and a 60× oil immersion TIRF objective with a numerical aperture of 1.49 (Olympus, Japan). Immersion Oil 23°C (#1261, Cargille, USA) was used. Samples were mounted onto the microscope in GLOX buffer (1.5% β-mercaptoethanol, 0.5% (v/w) glucose, 0.25 mg/ml glucose oxidase and 20 μg/ml catalase, 150 mM Tris-HCl pH 8.8), illumination at a laser-power density of 5.5 kW/cm2 using a 637 nm laser for Alexa Fluor 647 or a 561 nm laser at a laser-power density of 4.6 kW/cm^2^ for Cy3b. Images were collected with 20 ms acquisition time. Per probe (Cy3b or Alexa Fluor 647), 10,000 frames were acquired. Acquired raw data were localised using SRX (Bruker). Localisations were estimated by fitting single emitters to a 3D experimentally determined point spread function (PSF) under optimisation of maximum likelihood. The maximum number of localisation iterations performed before a given non-converging localisation was discarded, was set to 40. PSFs were interpolated using the B-spline method. Obtained localisations were filtered according to precision estimates based on the Thompson method, i.e., all localisations with localisation precision worse than 20 nm were excluded. Localisations were rendered as Gaussian distributions with a constant width of 20 nm. Alignment of colour channels and drift correction were performed in SRX using Tetraspeck beads (Thermo Fisher Scientific, T7279). In total, we acquired *d*STORM images from 2 to 4 independent experiments (2 for SynCAM 1/PSD-95; 3 for MPP2/PSD-95; 4 for SynCAM 1/MPP2). Supporting information figures (S1–S3 Figs) were prepared using the ScientiFig Plugin for Fiji/ImageJ.

#### Image analysis

To approximate the dimensions of those bracelet-like arrangements, these previously assessed synapses were assessed individually (S4 Fig). Using the “line tool” in FIJI/ImageJ, 3 measurements per synapse were taken for both channels (Cy3b and AF 647) independently and were averaged per synapse. Summarised data were acquired from 170 to 236 synapses in 6 to 8 images of 2 to 4 independent experiments. To assess the frequency of the described bracelet-like arrangement of SynCAM 1 and MPP2 clusters at the periphery of PSDs, we manually matched synapses from corresponding widefield and *d*STORM images based on the staining pattern of PSD-95 or MPP2 along a dendrite. Those dendritic spines, which were apparent in images acquired with both techniques, were then individually categorised whether SynCAM 1 and/or MPP2 (depending on the marker combination of the dual-colour stainings) exhibited a bracelet-like arrangement of protein clusters or not. Data were expressed as fraction of all synapses identified in both widefield and *d*STORM image (S4 Fig). Analysis was performed for approximately 57 synapses per image in 3 to 6 images per marker combination. The numerical data used in Fig 1 are included in S2 Data.

### SIM

#### Sample preparation

Primary hippocampal rat neurons (DIV21) stained for the presynaptic marker vGlut1 and the postsynaptic proteins PSD-95, SynCAM 1, and MPP2 as described above.

#### Image acquisition

Targets were selected based on the SynCAM 1 signal. Three-dimensional SIM images were acquired with the OMX V4 Blaze system (GE Healthcare, UK), using the 405 nm, 488 nm, 568 nm, and 647 nm laser lines, a 60× 1.42 N.A. oil objective (Olympus), an immersion oil with a refractive index of 1.518 and standard emission filters at 125 nm z-sectioning. Multicolour registration with an error below 40 nm was done using 100 nm fluorescent beads (TetraSpeck, T7284, Thermo Fisher Scientific). Images were acquired with the DeltaVision OMX acquisition software (GE Healthcare), and images were reconstructed with softWoRx (GE Healthcare). Parameters for the reconstruction can be found in the Supporting information (S1 Files). The quality of 3D SIM reconstructions was tested with SIMcheck [54]. The superresolution channels 642, 568, and 488 show a good signal-to-noise ratio and no signs of hexagonal artefacts. Fast Fourier transformed images uncover a high amount of information below the diffraction limit. Due to the limited brightness and stability of the Alexa Fluor 405 dye, the signal-to-noise ratio and resolution in the 405 nm channel were limited. We thus decided to use this channel only for vGlut1 as a reference for the segmentation of mature synapses and not as a structural superresolution readout. An image acquisition parameter log file is included in S1 Files.

### SIM image analysis

#### Segmentation

Image segmentation was performed in Arivis Vision 4D (Arivis, Munich, Germany). MPP2, SynCAM 1, and vGlut1 clusters were identified with histogram-based threshold procedures (Otsu’s and Yen’s method) after Gaussian filtering and background subtraction. PSD-95 clusters and their centres were identified with the built in “Blob Finder” tool, a combination of automatic seed finding and watershed segmentation (watershed level: 6.7, threshold: 4.5, expected blob diameter: 0.5 μm). Subsequently, such segmented PSD-95 clusters were further filtered for sphericity (>0.4) and volume (>000.5 μm^3^) to exclude unusual segmented clusters which likely only represent background. Further, the coexistence of MPP2, SynCAM 1, and vGlut1 staining within the same synapse (2 to 2.5 μm distance cutoff to the centre of the next PSD-95 cluster) was ensured by applying the built-in colocalisation tool. Only synapses within a manually determined and set range of planes (selected for the best signal intensity for PSD-95) were considered for further analysis. Staining for the presynaptic protein vGlut1 only served as a marker for mature synapses, but was not further included in the analysis due to the limited quality in the 405 channel. The Arivis pipeline is available as an XML file in the Supporting information (S1 Files). The segmentation was performed on 50 images of 3 independent experiments, and the whole dataset was subjected for further analysis.

To approximate the frequency with which synapses on our imaged neurons express all 3 proteins of interest (PSD-95, MPP2, and SynCAM 1), using the same segmentation pipeline, we examined 8 images from 2 independent experiments with regard to how the incorporation of colocalisation selectors MPP2 and SynCAM 1 affect the total number of segmented synapses.

#### Radial intensity profiles

A custom-written FIJI/ImageJ script (https://github.com/ngimber/RadialProfile3D) was used to calculate 3D radial intensity profiles around PSD-95 centres (segmentation from above). Radial intensity profiles were 0–1 normalised and averaged twice (per image and per experiment) using Python before plotting with Prism 7 (GraphPad, USA).

#### NN analysis in SIM datasets

NN analysis and randomisations were performed in Python using custom-written scripts (https://github.com/ngimber/NearestNeighborWorkflow_Synapses). NN distances between PSD-95, MPP2, and SynCAM 1 clusters were calculated based on the Arivis segmentations. Random controls were generated by randomly distributing spherical objects, representing PSD-95, MPP2, and SynCAM 1 clusters within a simplified spherical postsynapse with a diameter of 0.8 μm [17]. Randomised distributions were generated for each image using the object counts and volumes from the corresponding segmentation (10 simulation rounds per synapse, approximately 40.000 synapses from 50 images). Plotting was done with Prism 7 (GraphPad).

#### Object statistics

Object counts and sizes were obtained from the segmentation above (Arivis). Histograms (bin size = 15 nm) from cluster sizes were plotted in R+.

The numerical data used Fig 2 are included in S2 Data.

### Triple-colour *d*STORM imaging

#### Labelling of antibodies

CF 680 and CF 658 secondary antibodies were generated by incubating 100-μg IgG Fab fragments dissolved in in NaHCO_3_ (50 mM, pH 8.1) with a 5-fold excess of succinimidyl esters of fluorescent dye CF 680 or CF 568 dyes (Biotium, USA) in DMSO (10 μM) for 1 hour at RT under gentle agitation. Unbound dye was removed with Nap-5 Sephadex G-25 columns (GE Healthcare) and labelled antibody eluted from the column with PBS.

#### Sample preparation

Primary hippocampal rat neurons (DIV21) were stained for the postsynaptic proteins Homer1 as PSD marker, MPP2, and GABA_A_R α1 with respective primary antibodies as described above. Samples were then incubated with appropriate secondary antibody Fab fragments coupled to Alexa Fluor 647 (goat αMouse AF647, A21236, Invitrogen), CF 680 (goat αRabbit, custom labelled 111-007-008, Jackson ImmunoResearch) or CF 568 (goat αguinea pig, custom labelled 106-007-008, Jackson ImmunoResearch) for 1 hour at RT, followed by washes and application of fluorescent beads (100 nm, TetraSpeck, Life Technologies), 1:200 in 0.01% PLL/PBS.

#### Image acquisition

Triple-colour *d*STORM images were generated by using 2 channels acquired in SD-*d*STORM mode (simultaneous excitation at 642 nm of Alexa Fluor 647 (Invitrogen) and CF 680 (Biotium), [55,56]) and an additional channel (CF 568 (Biotium)) acquired in conventional *d*STORM mode The spectral de-mixing (SD-) mode has the advantage of being inherently free of any registration errors. The *d*STORM channel was registered towards the SD-*d*STORM channels using bead-based registration (100 nm, TetraSpeck, Invitrogen). Samples were imaged with the N-STORM super-resolution setup (Nikon Instruments, Japan) controlled by NIS-Elements (Nikon) and equipped with a sCMOS camera (Prime 95B, Photometrics, USA), a 100x oil-immersion objective (NA = 1.49; Nikon), an additional 1.5x optovar lens (Nikon) and an emission splitter (OptoSplit III; Cairn Research, UK) in the emission path for the SD-mode. The emission splitter was equipped with a dichroic mirror (700-DCXXR; AHF Analysentechnik, Germany) for spectral demixing of AF 647 and CF 680. An autofocus system (PerfectFocus II; Nikon) was used to prevent focal drift. Lateral drift was minimised by stabilising the temperature at 26°C with an incubator (Okolab, Italy) and corrected using immobilised beads (100 nm, Tetraspeck, Invitrogen) as fiduciary marks. Samples were mounted in *d*STORM imaging buffer: 0.5 mg/ ml glucose oxidase (Sigma-Aldrich, USA), 40 mg/ ml catalase (Roche, Switzerland), 10% (w/ v) glucose, 100 mM MEA (β-mercaptoethylamine; Sigma-Aldrich) in PBS. Samples were illuminated with a 642 nm laser diode at about 3.5 kW/cm^2^ for the SD-mode and with a 561 nm laser at 1.2 kW/cm^2^ for the *d*STORM-mode. Typically, we recorded 15,000 frames with an exposure time of 30 ms for all channels. Image acquisition parameter log files are included in S1 Files.

#### Single molecule localisation and drift correction

The open source Fiji/ImageJ [52] plugin ThunderSTORM1.3 [57] was used to localise single molecule blinking events. Specifically, we used the “integrated Gaussian” and the “weighted least squares” functions of ThunderSTORM with a fitting radius of 4 pixels (292 nm) and an initial sigma of 1.5 pixels (110 nm) to localise the events. Further details are provided in the parameters files (S1 Files). Localisations were rendered as Gaussian distributions (FWHM = 20 nm). TetraSpeck beads were used for drift-correction and alignment of the 568 channel to the de-mixed 647/680 channels.

#### Spectral de-mixing

We used the recently published open source software tool SD-Mixer2 ([58]; https://github.com/gtadeus/sdmixer2) for pairing and colour assignment to Alexa 648/CF 680 localisations. ThunderSTORM localisation files were converted into the SD-Mixer file format (custom Python script available on GitHub: https://github.com/ngimber/Converter_ThunderSTORM_SDmixer). Parameter files and binary masks for colour separation can be found in the Supporting information (S1 Files and S13 Fig).

#### Cluster detection in triple-colour dSTORM datasets

Clusters were automatically detected with the DBSCAN (Density-based spatial clustering of applications with noise) approach using the Python Package Scikit-learn 0.22.2 (Parameters: ε = 20 nm, min 5 points) [59]. Histograms from 26 images (from 3 independent experiments) were averaged. Plotting was done with Prism 7 (GraphPad).

#### NN analysis in dSTORM datasets

NN analysis and randomisation were performed in Python using a custom-written script. Clusters were detected, as described above. NN distances were calculated between MPP2 and GARBAR cluster centres. Random controls were generated for each image by introducing a toroidal shift of 20 nm to the MPP2 channel. Histograms from 26 images (from 3 independent experiments) were averaged. Plotting was done with Prism 7 (GraphPad).

The numerical data used in Fig 8 are included in S2 Data.

### Cell culture and transfection

HEK293T and CHL V79 cells were maintained in low-glucose DMEM supplemented with 10% FCS, 1,000 U/ml penicillin/streptomycin and 2 mM L-glutamine in a humidified incubator at 37 °C with 5% CO_2_. Transfections were performed using Lipofectamine 2000 (Invitrogen) and desired DNA constructs diluted in Opti-MEM (Gibco).

### DNA and shRNA constructs

N-terminal FLAG-tagged MPP2 and PSD-95 were cloned as described before [13,60]. Full-length mouse MPP2 (NM_016695) was cloned into pEGFP-C1, to obtain N-terminal EGFP-tagged EGFP-MPP2. Using NheI and EcoRI restriction sites this was further transferred into f(w)syn lentiviral vector provided by VCF. Full-length rat SynCAM 1 (NM_001012201.1) was synthesised as described before [13]. Clover-PSD-95. Expression constructs for Flag-tagged full-length rat proteins were generated by cloning Arhgef2 (NM_001012079), Ppp3ca (NM_017041), and Farp1 (NM_001107287) into pCMV-Tag 2A. HA-Gnao1 (NM_017327) was constructed by PCR with a forward primer that encodes the HA tag and cloned with NotI and SalI into pCMV-Tag 2A.

GFP-PIPK1 gamma 90 was a gift from Pietro De Camilli (Addgene plasmid # 22299; http://n2t.net/addgene:22299; RRID: Addgene_22299) [61]. GABA (A) receptor subunit a1SE was a gift from Tija Jacob and Stephen Moss (Addgene plasmid # 49168; http://n2t.net/addgene:49168; RRID: Addgene_49168). GABA(A) receptor subunit a2SE was a gift from Tija Jacob and Stephen Moss (Addgene plasmid # 49169; http://n2t.net/addgene:49169; RRID:Addgene_49169) [62]. GABA(A) receptor subunit B3SE was a gift from Tija Jacob and Stephen Moss (Addgene plasmid # 49171; http://n2t.net/addgene:49171; RRID:Addgene_49171) [63].

The bacterial expression construct GST-MPP2-SH3-GK was generated by cloning the fragment encoding amino acids 220–552 (SH3GK) of mouse MPP2 into pGEX-6P-1 (GE Healthcare). GST-PSD-95-SH3GK was generated as described before [23]. A fragment corresponding to amino acids 403–724 of PSD-95 was cloned into pGEX-6P-1.

MPP2 knockdown sequences used in this study were cloned into pSUPER and tested for knockdown efficiency and specificity using MPP family protein constructs transfected into CHL V79 cells. DNA sequence 5′ GGT CTA GAT CCC ACG TTT Att caa gag aTA AAC GTG GGA TCT AGA CC 3′, which results in transcription of a short hairpin RNA targeting the L27-PDZ domain linker region of the MPP2 protein, was cloned into the pSUPER vector according to the manufacturer’s instructions. It effectively and selectively hindered MPP2 protein expression in CHL V79 cells, and the same sequence was subsequently cloned into the f(w)syn lentiviral expression vector provided by Dr. Thorsten Trimbuch (Charité Virus Core Facility, VCF). Efficient knockdown of endogenous MPP2 was validated in both western blot and immunofluorescence experiments (see S8 Fig). For control, 2 scrambled shRNA constructs (f(w)syn-Scrbl-Clathrin and f(w)syn-Scrbl-Renilla) in the same lentiviral expression vector were used.

### Co-immunoprecipitation

HEK293T cells were harvested 18 to 20 hours after transfection with a cell scraper and cell lysates obtained with 30 gauge syringe needle strikes in immunoprecipitation buffer (50 mM Tris pH 7.4; 100 mM NaCl; 1 mM EDTA, 1% Triton-X or 0.1% NP-40; supplemented with Complete Mini protease inhibitors, Roche). Cell lysates were cleared by centrifugation (3× 10 minutes at 20,000 *g* at 4°C), and supernatants were incubated with 2 μg αGFP (mouse, 75–131, NeuroMab) or normal mouse IgG for 3 hours on a rotator (10 rpm) at 4°C. Pull-down was performed with 30 μl protein-G-agarose bead slurry (Roche) for 1 hour at 4°C under gentle agitation, followed by 3 washes with IP buffer and final analysis by western blot.

### Western blotting

Lysates and beads were boiled in 2x SDS-sample buffer (8% SDS, 40% glycerol, 0.25 M Tris pH 6.8, 20% β-mercaptoethanol) for 5 minutes, separated on a 10% SDS-PAGE, and blotted onto PVDF membranes (Roche). Membranes were blocked with 5% skim milk/PBST. Primary antibodies were diluted in the blocking buffer and applied over night at 4°C, followed by 3 washes with PBST and 1 hour incubation with appropriate secondary antibodies. HRP signals were detected using Western Lightning chemiluminescent substrates (Perkin Elmer, USA) with a luminescent image analyser (ImageQuant LAS4000, GE Healthcare).

**Primary antibodies:** αGABA_A_R α1 (mouse, 75–136, NeuroMab), αFlag (mouse, F1804, Sigma), αFlag-HRP (mouse, A8592, Sigma), αGFP (chicken, ab13970, Abcam), αGST (mouse, 75–148, NeuroMab), and αHA (rabbit, H6928, Sigma).

**Secondary antibodies:** αMouse-HRP (115-035-003, Dianova), αMouse-native-IgG (Veriblot, 131368, Abcam), αRabbit-HRP (111-035-003, Dianova), and αChicken-HRP (ab6753, Abcam).

### Crude synaptosome fraction preparation

For immunoprecipitation using brain lysates, adult Wistar rats were administered isofluorane anaesthesia prior to decapitation and reported under permit T0280/10 (LaGeSO). The brains were removed and rinsed in ice-cold PBS. Brains were immediately frozen and stored at −80°C until use. Brains (approximately 2 g) were then thawed on ice, minced with a scalpel and homogenised in 20 ml Syn-PER Synaptic Protein Extraction Reagent (Thermo Science, USA) according to the manufacturer’s protocol. For co-immunoprecipitation, the resulting crude synaptosome fraction was then resuspended in IP buffer (50 mM Tris pH 7.4; 100 mM NaCl; 1 mM EDTA, 1% Triton-X; supplemented with Complete Mini protease inhibitors, Roche) and cleared by 3x centrifugation at 20,000 *g*. For GST pull-down, the pellet was solubilised in 10 ml 1% Triton-X/PBS.

### GST pull-down

GST-SH3-GK domain constructs of PSD-95 and MPP2 were expressed in *Escherichia coli* BL21 DE3 and purified according to the manufacturer’s manual (GST Gene Fusion System, GE Healthcare). Moreover, 30 μl of glutathione agarose (Pierce, USA) was loaded with GST-SH3-GK proteins (PSD-95 and MPP2) and incubated for 3 hours with protein lysate from crude synaptosomes. The beads were washed 3 times with PBS/1% Triton X-100 and eluted from the matrix by incubation with SDS sample buffer for 5 minutes at 95°C.

### Sample preparation and liquid chromatography–mass spectrometry (LC–MS)

Proteins from 2 technical replicates were separated by SDS-PAGE (10% Tricine-SDS-PAGE). Coomassie-stained lanes were cut into 12 slices and in-gel protein digestion and ^16^O/^18^O-labelling was performed as described previously [64,65]. In brief, corresponding samples (PSD-95 and MPP2) were incubated overnight at 37°C with 50 ng trypsin (sequencing grade modified, Promega) in 25 μl of 50 mM ammonium bicarbonate in the presence of heavy water (Campro Scientific GmbH, 97% ^18^O) and regular ^16^O-water, respectively. Isotope labels were switched between the 2 replicates. To prevent oxygen back-exchange by residual trypsin activity, samples were heated at 95°C for 20 minutes. After cooling down, 50 μl of 0.5% trifluoroacetic acid (TFA) in acetonitrile was added to decrease the pH of the sample from approximately pH 8 to approximately pH 2. Afterwards, corresponding heavy- and light-isotope labelled samples were combined and peptides were dried under vacuum. Peptides were reconstituted in 10 μl of 0.05% TFA, 2% acetonitrile in water, and 6.5 μl were analysed by a reversed-phase nano liquid chromatography system (Ultimate 3000, Thermo Fisher Scientific) connected to an Orbitrap Velos mass spectrometer (Thermo Fisher Scientific). Samples were injected and concentrated on a trap column (PepMap100 C18, 3 μm, 100 Å, 75 μm i.d. × 2 cm, Thermo Fisher Scientific) equilibrated with 0.05% TFA, 2% acetonitrile in water. After switching the trap column inline, LC separations were performed on a capillary column (Acclaim PepMap100 C18, 2 μm, 100 Å, 75 μm i.d. × 25 cm, Thermo Fisher Scientific) at an eluent flow rate of 300 nl/min. Mobile phase A contained 0.1% formic acid in water, and mobile phase B contained 0.1% formic acid in acetonitrile. The column was preequilibrated with 3% mobile phase B followed by an increase of 3% to 50% mobile phase B in 50 minutes. Mass spectra were acquired in a data-dependent mode using a single MS survey scan (m/z 350 to 1,500) with a resolution of 60,000 in the Orbitrap, and MS/MS scans of the 20 most intense precursor ions in the linear trap quadrupole. The dynamic exclusion time was set to 60 seconds and automatic gain control was set to 1 × 10^6^ and 5,000 for Orbitrap-MS and LTQ-MS/MS scans, respectively.

### Proteomic data analysis

Identification and quantification of ^16^O/^18^O-labelled samples was performed using the Mascot Distiller Quantitation Toolbox (version 2.7.1.0, Matrix Science). Data were matched against the SwissProt protein sequence database using the taxonomy rattus (August 2017 release with 7,996 protein sequences). Sequences of the employed protein constructs and the sequence of the GST tag were manually added to the database. A maximum of 2 missed cleavages was tolerated, and the mass tolerance of precursor and fragment ions was set to 15 ppm and 0.35 Da, respectively. Methionine oxidation, acetylation (protein N-terminus), propionamide (Cysteine), and carboxyl-terminal ^18^O_1_- and ^18^O_2_-isotope labelling were used as variable modifications. A significance threshold of 0.05 at the peptide level was used based on decoy database searches. At the protein level, a minimum of 2 quantified peptides was set as a threshold. Protein ratios were calculated from the intensity-weighted average of all corresponding peptide ratios. The protein ratio of GST was used for normalisation of protein ratios. Only proteins that were quantified in both replicates with a geometric standard deviation of <2 were considered. Known contaminants (e.g., keratins) and the bait proteins were removed from the protein output table.

### Electrophysiology

Primary hippocampal neurons were prepared and plated onto coverslips as described earlier. At DIV3 neurons were infected with lentivirus transferring either control or shRNA to knockdown MPP2 expression or at DIV 10 with an EGFP-MPP2 construct for overexpression. Infected neurons were left undisturbed until measuring. At DIV 17–21 coverslips were transferred into recording chamber filled with extracellular solution (140 mM NaCl, 2.4 mM KCl, 10 mM Hepes, 10 mM glucose, 4 mM CaCl_2_, 2 mM MgCl_2_). Neurons exhibiting a pyramidal-like shaped soma were recorded with the patch-clamp technique in voltage-clamp mode (clamped at −70 mV) using a KCl-based intracellular solution (136 mM KCl, 17.8 mM Hepes, 1 mM EGTA, 0.6 mM MgCl_2_, 5 mM MgATP, 12 mM Na-Phosphocreatine, 50 U/ml Phosphocreatine kinase, 0.3 mM Na_2_GTP). Recorded traces were considered for analysis when Rs < 20 Ω and I_hold_ < 200 pA. In order to record exclusively spontaneous mIPSCs neurons were treated with TTX (1 μM), AP5 (25 μM) and NBQX (10 μM) during recordings. Signals were detected automatically using IGOR Pro with the plugin Neuromatics and subsequently manually sorted by visual inspection. Cumulative distributions of mIPSC IEIs and individual event amplitudes were analysed using an equal number of events per cell per condition to prevent overrepresentation of single neurons.

The numerical data used in all figures are included in S2 Data.

## Supporting information

S1 Fig Dual-colour *d*STORM images of SynCAM 1 and PSD-95. E18 rat primary hippocampal neurons were fixed at DIV21 and stained for endogenous SynCAM 1 (magenta) and PSD-95 (cyan) proteins with Alexa Fluor 647 and Cy3b-coupled secondary antibodies. Protein localisations were filtered according to the Thompson method, i.e., all localisations with accuracy below 20 nm were excluded. Scale bars: 1 μm. DIV, days in vitro; *d*STORM, direct stochastic optical reconstruction microscopy; PSD, postsynaptic density.
(TIF)

S2 Fig Dual-colour *d*STORM images of MPP2 and PSD-95. E18 rat primary hippocampal neurons were fixed at DIV21 and stained for endogenous MPP2 (magenta) and PSD-95 (cyan) proteins with Alexa Fluor 647 and Cy3b-coupled secondary antibodies. Protein localisations were filtered according to the Thompson method, i.e., all localisations with accuracy below 20 nm were excluded. Scale bars: 1 μm. DIV, days in vitro; *d*STORM, direct stochastic optical reconstruction microscopy; MPP2, membrane protein palmitoylated 2; PSD, postsynaptic density.
(TIF)

S3 Fig Dual-colour *d*STORM images of SynCAM 1 and MPP2. E18 rat primary hippocampal neurons were fixed at DIV21 and stained for endogenous SynCAM 1 (magenta) and MPP2 (cyan) proteins with Alexa Fluor 647 and Cy3b-coupled secondary antibodies. Protein localisations were filtered according to the Thompson method, i.e., all localisations with accuracy below 20 nm were excluded. Scale bars: 1 μm. DIV, days in vitro; *d*STORM, direct stochastic optical reconstruction microscopy; MPP2, membrane protein palmitoylated 2.
(TIF)

S4 Fig Prevalence of synaptic bracelet-like SynCAM 1 and MPP2 cluster arrangements. SynCAM 1 and MPP2 clusters are arranged in a bracelet-like manner in the majority of synapses. Exemplary image to illustrate the manual assessment of the frequency of bracelet-like arrangement of SynCAM 1 and MPP2 at synapses. In corresponding sections of widefield (left column) and dual-colour *d*STORM images (right column), synaptic structures that were captured with both techniques were identified (white boxes, second row) and then individually assessed whether SynCAM 1 and/or MPP2 (magenta) protein clusters are arranged to form a bracelet-like structure (white circles, third row) around PSD-95 (cyan), if applicable. Scale bars: overview = 5 μm; detail = 1 μm; *d*STORM, direct stochastic optical reconstruction microscopy; MPP2, membrane protein palmitoylated 2; PSD, postsynaptic density.
(TIF)

S5 Fig NN analysis of SynCAM 1 and MPP2 protein clusters derived from 3D SIM images. NN distances from SynCAM 1 to the nearest MPP2 cluster were calculated between cluster centres (upper panel, grey bars) and cluster surfaces (lower panel). Dashed lines represent the upper and lower envelopes of CSR. CSR was calculated by randomly distributing MPP2 within the volume and SynCAM 1 on the surface of spheres of 0.8 μm diameter as indicated by the grey dotted line (mean ± SEM, 95% confidence interval, 10 simulations per synapse, *N* = 3 independent experiments, approximately 40.000 synapses from 50 images). CSR, complete spatial randomness; MPP2, membrane protein palmitoylated 2; NN, nearest neighbour; SIM, structured illumination microscopy.
(TIF)

S6 Fig Validation of novel MPP2 interaction partners by co-immunoprecipitation. **(a)** EGFP-tagged Pip5k1c was coexpressed with FLAG-tagged MPP2 in HEK293T cells. EGFP-Pip5k1c was precipitated with αGFP antibody or normal IgG as negative control and analysed by western blot with αFLAG and αGFP antibodies. An additional IgG control lane is marked with an asterisk. **(b)** FLAG-tagged Farp1 was overexpressed together with EGFP-tagged MPP2 and copurifies with αGFP pull-down, as opposed to normal IgG as negative control. Co-immunoprecipitation was detected by western blot probing with αFLAG and αGFP antibodies. **(c)** Copurification of FLAG-tagged Ppp3ca (a Calcineurin subunit) overexpressed together with EGFP-MPP2 after αGFP pull-down or normal Ms IgG as negative control, detected by western blot with αFLAG and αGFP antibodies. **(d)** Co-immunoprecipitation of FLAG-tagged Arhgef2 together with EGFP-MPP2 after pull-down with αGFP antibody or IgG control, as detected by western blot using αFLAG and αGFP antibodies. **(e)** HA-tagged Gnao1 was overexpressed together with EGFP-tagged MPP2 in HEK293T cells. Upon pull-down with Ms αGFP antibody or normal Ms IgG, Gano1 copurification and GFP pull-down control were detected by western blot with αHA and αGFP antibodies. MPP2, membrane protein palmitoylated 2.
(TIF)

S7 Fig Knockdown of endogenous MPP2. **(a)** Cotransfection in CHL V79 cells of MPP1, MPP2, MPP3, MPP5, and MPP6 expression constructs together with shRNA targeting MPP2 or control shRNA, respectively, leads to loss of expression of MPP2 together with shRNA, confirming efficacy and specificity of the selected shRNA sequence. **(b)** The same sequence introduced to cultured hippocampal neurons at DIV 3 with lentivirus-mediated knockdown, successfully abolishes expression of endogenous MPP2 as demonstrated by western blot analysis of whole cell lysates harvested at DIV 21 probed with aMPP2 antibody. DIV, days in vitro; MPP2, membrane protein palmitoylated 2.
(TIF)

S8 Fig NN analysis of GABA_A_R α1 and MPP2 protein cluster distances derived from 3-colour *d*STORM images. NN analysis of GABA_A_R α1 and MPP2 protein clusters after DBSCAN. NN distances were calculated from the cluster centres. Closest clusters of MPP2 to GABA_A_R α1 were analysed (grey bars). Dashed lines represent the random control by toroidal shift. Note the close association of both clusters (approximately 10 nm distance between both centres), which is well below the cluster sizes (20 and 40 nm, see Fig 6B) and shifted after randomisation. Mean ± SEM; *n* = 26 images from *N* = 3 independent experiments; *d*STORM, direct stochastic optical reconstruction microscopy; GABA, γ-aminobutyric acid; MPP2, membrane protein palmitoylated 2; NN, nearest neighbour.
(TIF)

S9 Fig Full-length blots for Fig 4. (TIF)

S10 Fig Full-length blots for S6 Fig. (TIF)

S11 Fig SIM image segmentation for PSD-95. Image segmentation pipeline of PSD-95 signal implemented in Arivis Vision 4D. Top row left: maximum projected overview. Top row: single image plane overview. White box indicates location of detail view. Scale bar: 10 μm. Top right: single plane 4-colour detail view used to illustrate the segmentation steps below. Scale bar: 5 μm. PSD, postsynaptic density; SIM, structured illumination microscopy.
(TIF)

S12 Fig SIM image segmentation for MPP2, SynCAM 1, and vGlut1. Image segmentation of MPP2, SynCAM 1, and vGlut1 signal implemented in Arivis Vision 4D. Top row left: maximum projected overview. Top row: single image plane overview. White box indicates location of detail view. Scale bar = 10 μm. Top right: single plane 4-colour detail view used to illustrate the segmentation steps below. Scale bar = 5 μm. Lower panel, left column: MPP2 segmentation; middle column: SynCAM 1 segmentation, right column: vGlut1 segmentation. Respective image channels were segmented by applying Otsu’s and Yen’s auto-threshold methods and selecting segments within 2 μm around a PSD-95 cluster (blue). MPP2, membrane protein palmitoylated 2;PSD, postsynaptic density; SIM, structured illumination microscopy.
(TIF)

S13 Fig Associated content to Fig 8. Colour separation masks for spectral de-mixing (SD)-*d*STORM. *d*STORM, direct stochastic optical reconstruction microscopy.
(TIF)

S14 Fig Full-length blots for S7 Fig. (TIF)

S1 Data Underlying source data for Fig 3C. (XLSX)

S2 Data Spreadsheet containing the underlying numerical data and statistical analysis for Figure panels 1d, 2bcd, 5bcef, 7abc, 8bc, S5, and S8 in separate sheets. (XLSB)

S1 Files *.zip archive containing image acquisition and analysis parameter files. (ZIP)

## Abbreviations

AMPA: α-amino-3-hydroxy-5-methyl-4-isoxazolepropionic acid
DIV: days in vitro
*d*STORM: direct stochastic optical reconstruction microscopy
GABA: γ-aminobutyric acid
IEI: interevent interval
LC–MS: liquid chromatography–mass spectrometry
MAGUK: membrane-associated guanylate kinase
mIPSC: miniature inhibitory postsynaptic current
MPP2: membrane protein palmitoylated 2
NMDAR: N-methyl-D-aspartate receptor
NN: nearest neighbour
PSD: postsynaptic density
PSF: point spread function
RT: room temperature
SIM: structured illumination microscopy
VCF: Virus Core Facility
