# Supplementary figures and images for: The synaptic scaffold protein MPP2 interacts with GABAA receptors at the periphery of the postsynaptic density of glutamatergic synapses
Source: PLoS Biol. 2022 Mar 21;20(3):e3001503. doi: 10.1371/journal.pbio.3001503 (PMC8970474; doi:10.1371/journal.pbio.3001503)

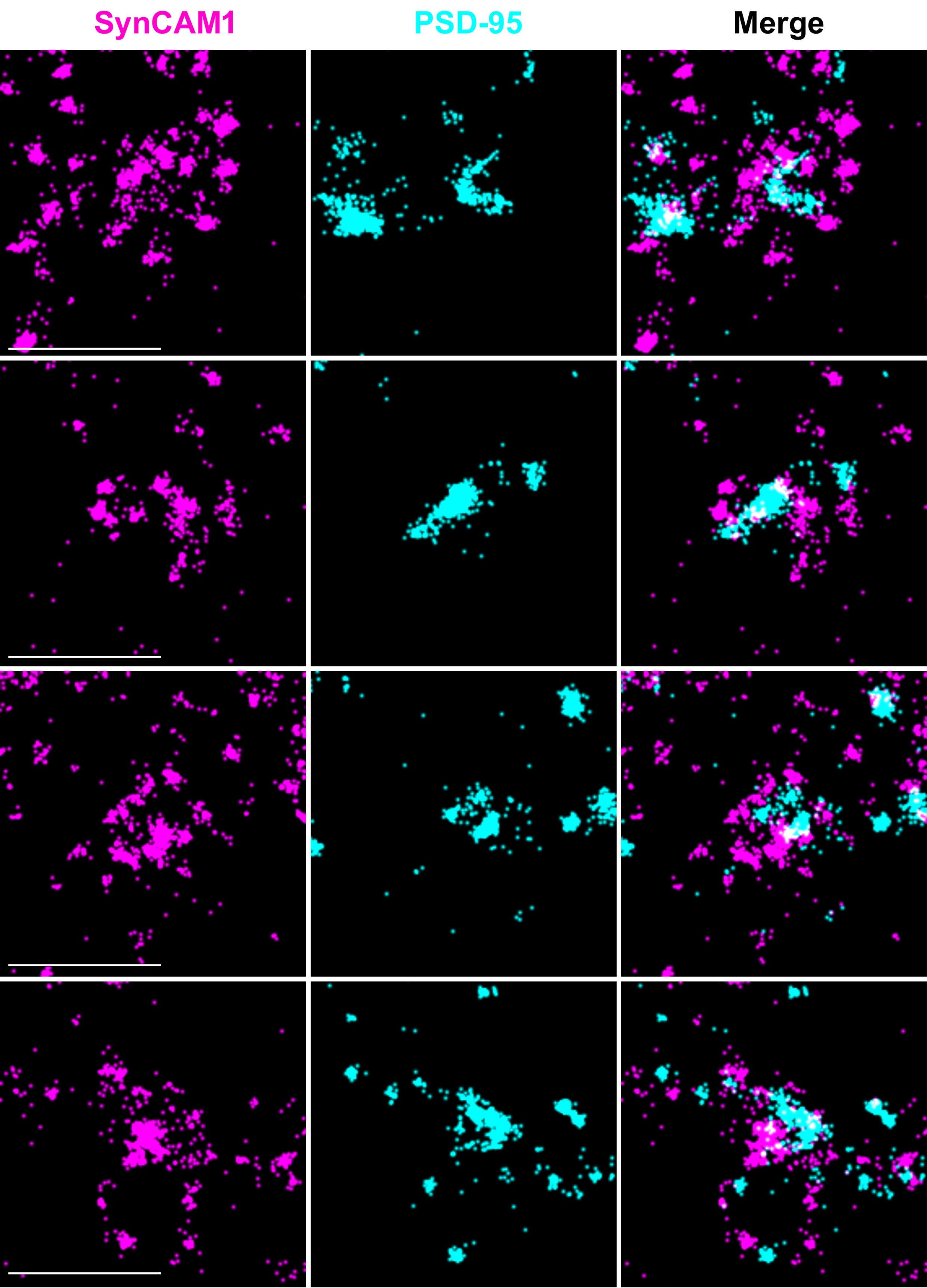

Supplement: S1 Fig — E18 rat primary hippocampal neurons were fixed at DIV21 and stained for endogenous SynCAM 1 (magenta) and PSD-95 (cyan) proteins with Alexa Fluor 647 and Cy3b-coupled secondary antibodies. Protein localisations were filtered according to the Thompson method, i.e., all localisations with accuracy below 20 nm were excluded. Scale bars: 1 μm. DIV, days in vitro; dSTORM, direct stochastic optical reconstruction microscopy; PSD, postsynaptic density. (TIF) [file pbio.3001503.s001.tif]

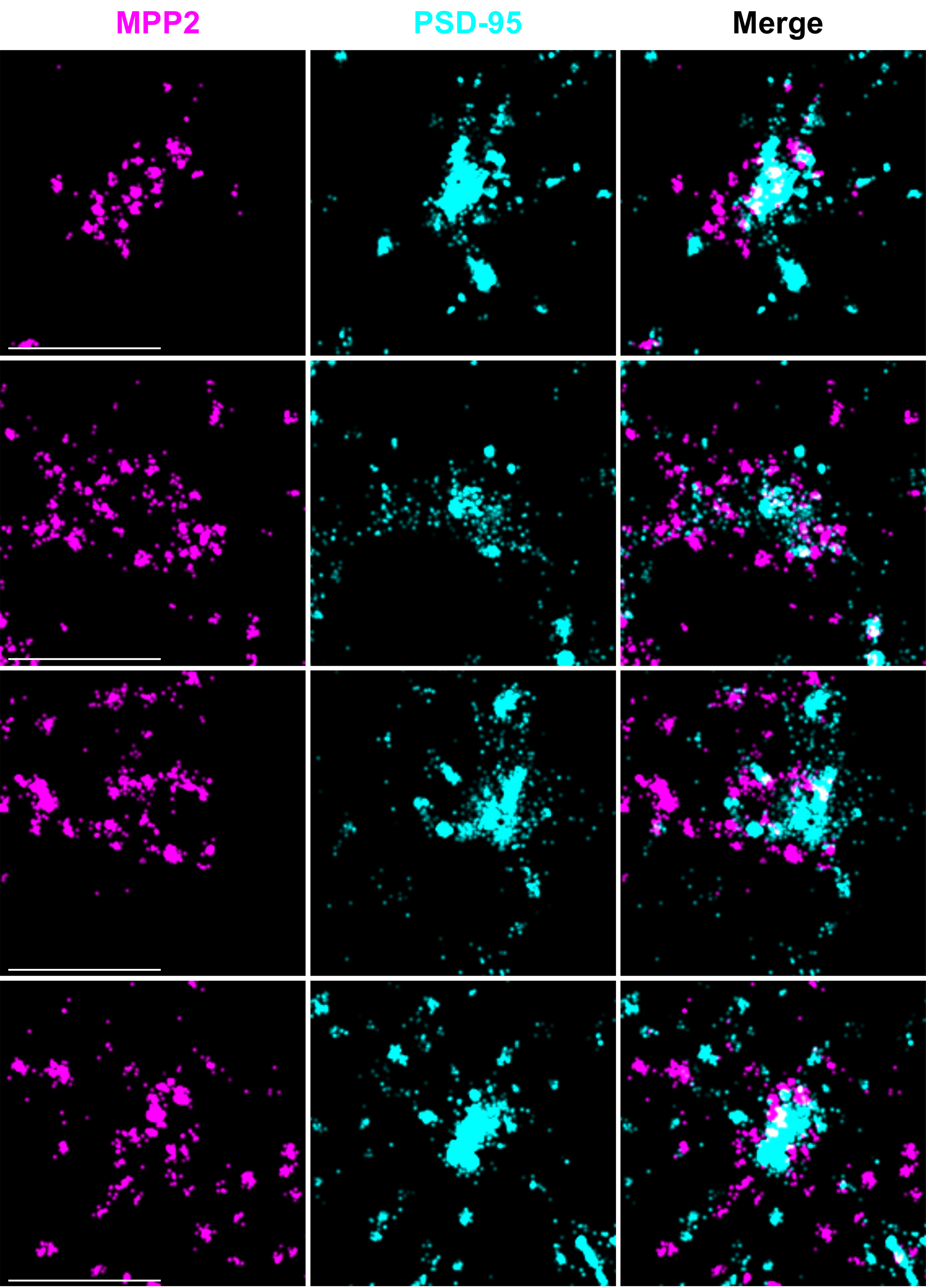

Supplement: S2 Fig — E18 rat primary hippocampal neurons were fixed at DIV21 and stained for endogenous MPP2 (magenta) and PSD-95 (cyan) proteins with Alexa Fluor 647 and Cy3b-coupled secondary antibodies. Protein localisations were filtered according to the Thompson method, i.e., all localisations with accuracy below 20 nm were excluded. Scale bars: 1 μm. DIV, days in vitro; dSTORM, direct stochastic optical reconstruction microscopy; MPP2, membrane protein palmitoylated 2; PSD, postsynaptic density. (TIF) [file pbio.3001503.s002.tif]

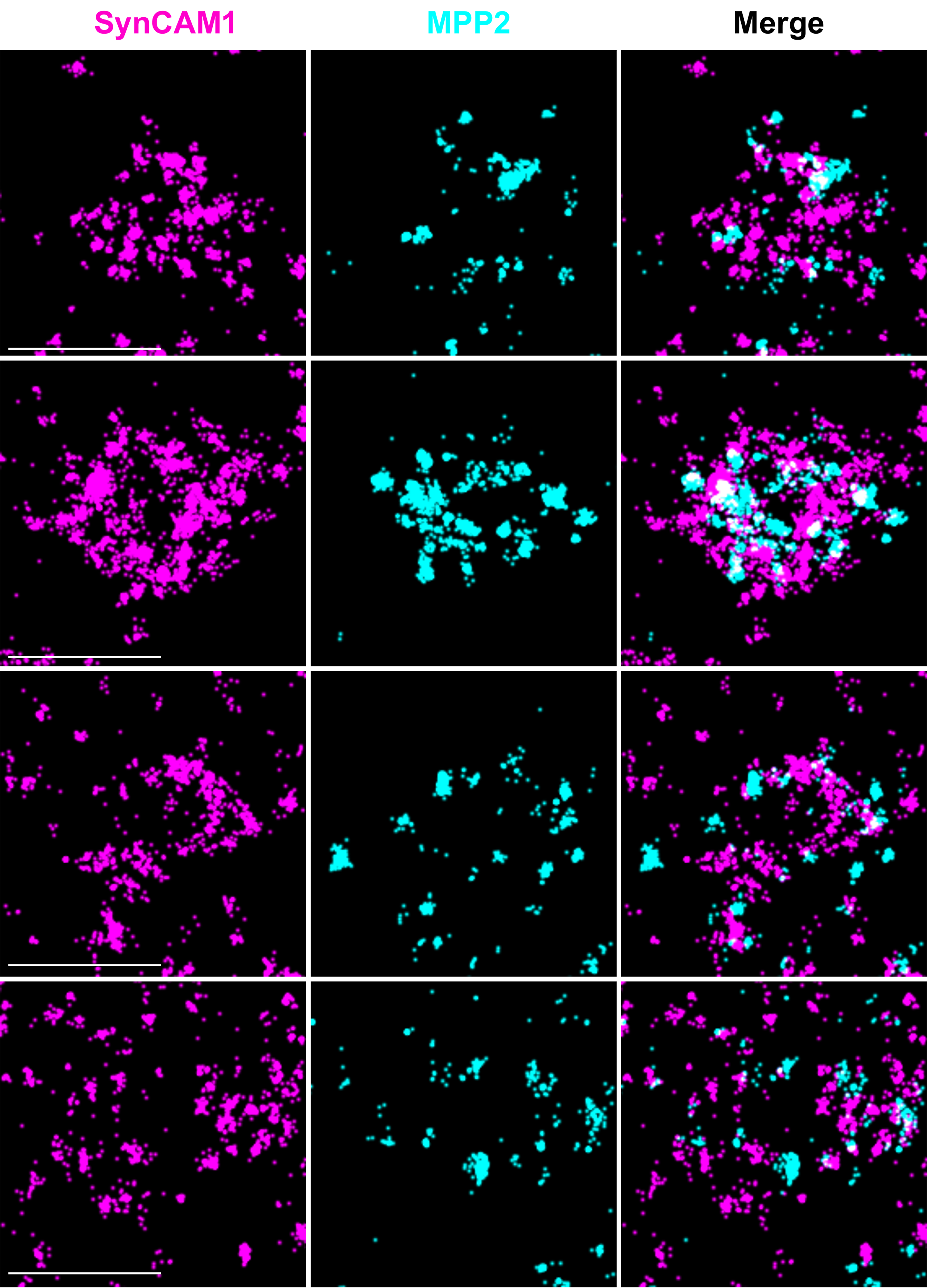

Supplement: S3 Fig — E18 rat primary hippocampal neurons were fixed at DIV21 and stained for endogenous SynCAM 1 (magenta) and MPP2 (cyan) proteins with Alexa Fluor 647 and Cy3b-coupled secondary antibodies. Protein localisations were filtered according to the Thompson method, i.e., all localisations with accuracy below 20 nm were excluded. Scale bars: 1 μm. DIV, days in vitro; dSTORM, direct stochastic optical reconstruction microscopy; MPP2, membrane protein palmitoylated 2. (TIF) [file pbio.3001503.s003.tif]

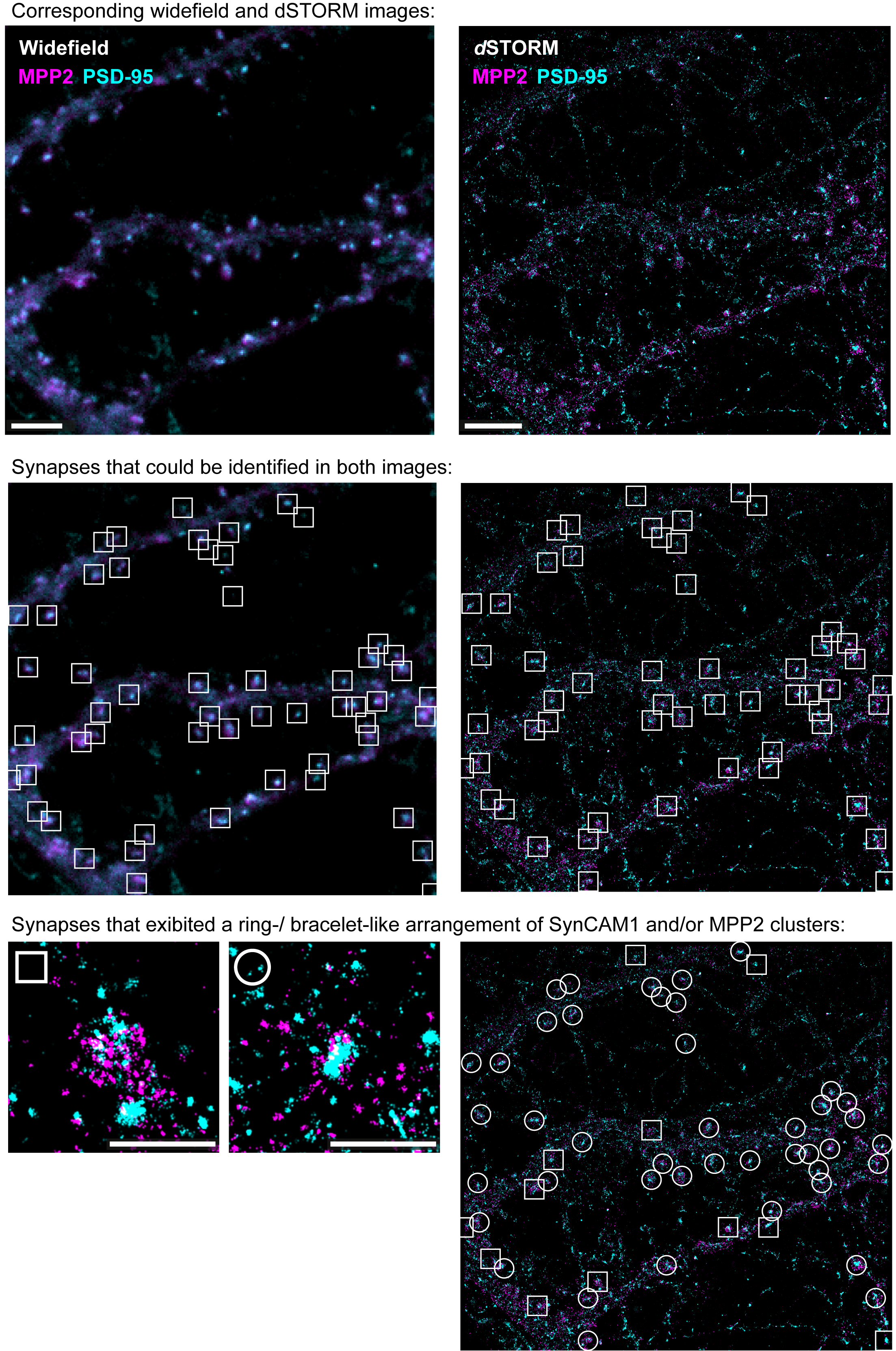

Supplement: S4 Fig — SynCAM 1 and MPP2 clusters are arranged in a bracelet-like manner in the majority of synapses. Exemplary image to illustrate the manual assessment of the frequency of bracelet-like arrangement of SynCAM 1 and MPP2 at synapses. In corresponding sections of widefield (left column) and dual-colour dSTORM images (right column), synaptic structures that were captured with both techniques were identified (white boxes, second row) and then individually assessed whether SynCAM 1 and/or MPP2 (magenta) protein clusters are arranged to form a bracelet-like structure (white circles, third row) around PSD-95 (cyan), if applicable. Scale bars: overview = 5 μm; detail = 1 μm; dSTORM, direct stochastic optical reconstruction microscopy; MPP2, membrane protein palmitoylated 2; PSD, postsynaptic density. (TIF) [file pbio.3001503.s004.tif]

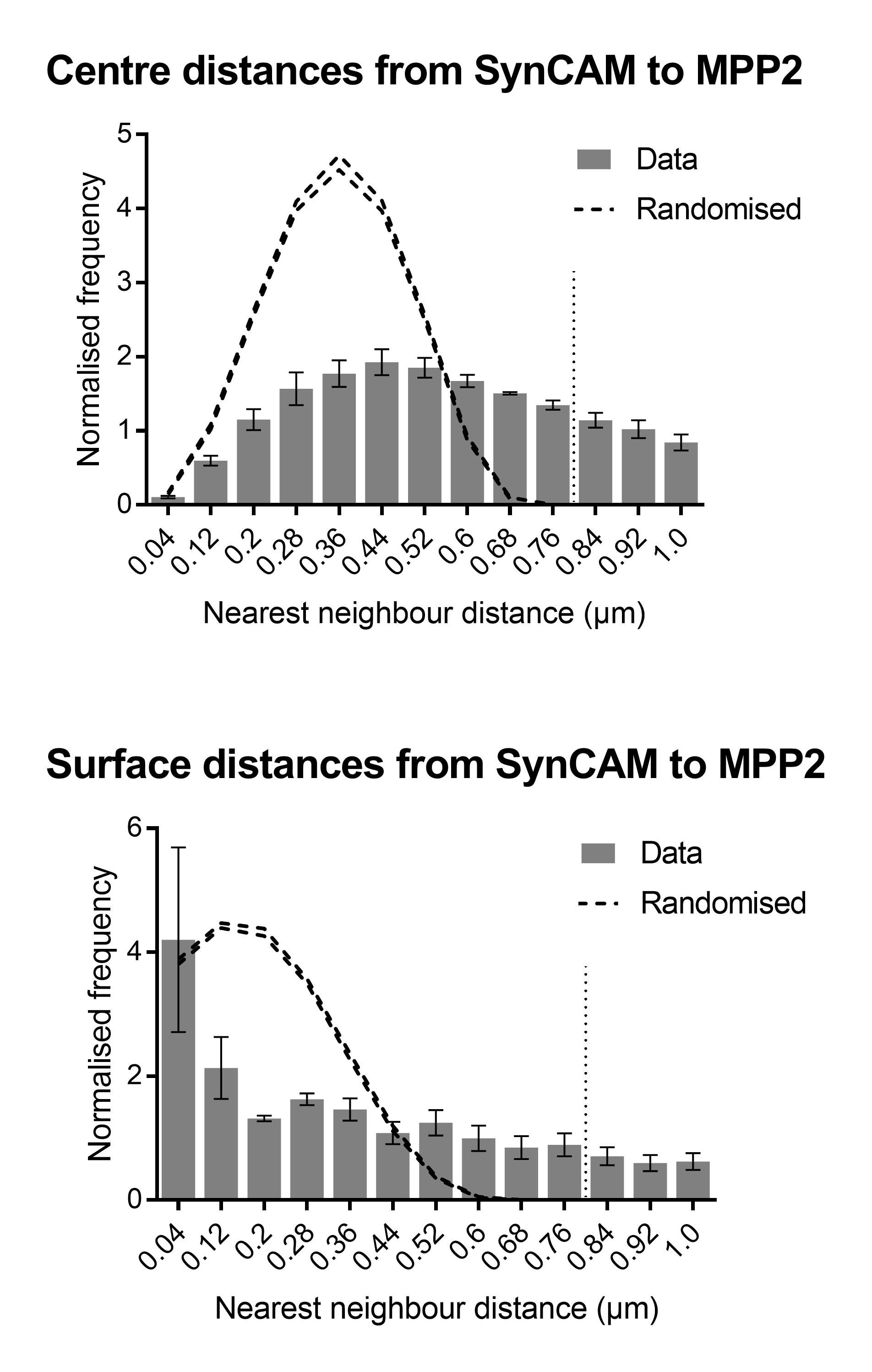

Supplement: S5 Fig — NN distances from SynCAM 1 to the nearest MPP2 cluster were calculated between cluster centres (upper panel, grey bars) and cluster surfaces (lower panel). Dashed lines represent the upper and lower envelopes of CSR. CSR was calculated by randomly distributing MPP2 within the volume and SynCAM 1 on the surface of spheres of 0.8 μm diameter as indicated by the grey dotted line (mean ± SEM, 95% confidence interval, 10 simulations per synapse, N = 3 independent experiments, approximately 40.000 synapses from 50 images). CSR, complete spatial randomness; MPP2, membrane protein palmitoylated 2; NN, nearest neighbour; SIM, structured illumination microscopy. (TIF) [file pbio.3001503.s005.tif]

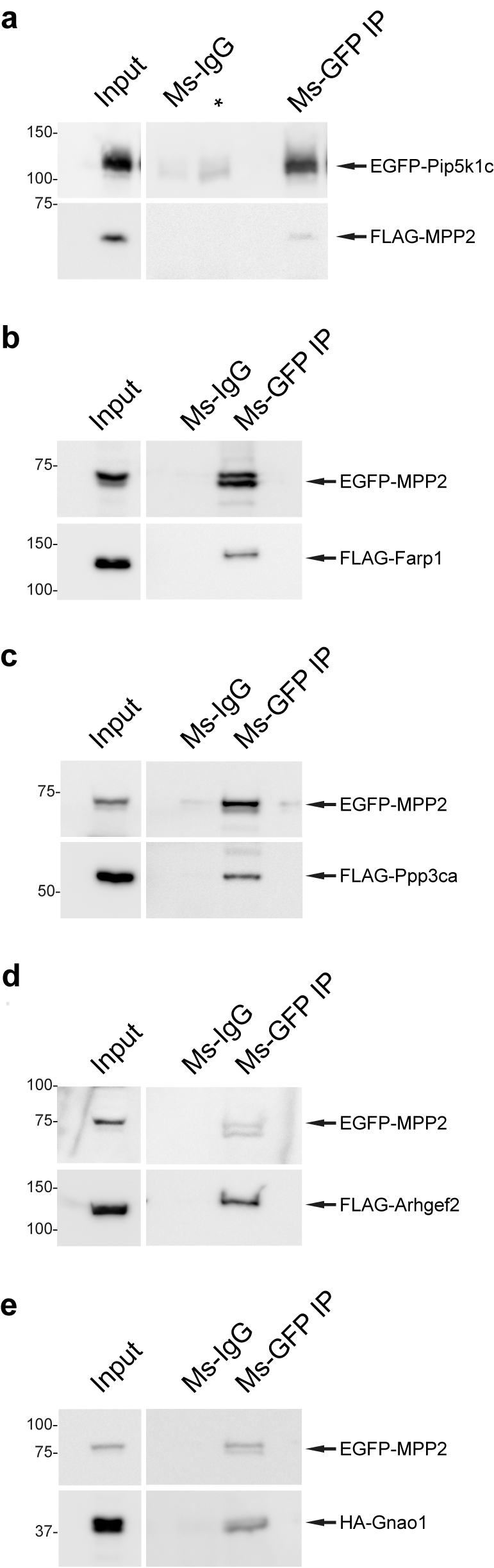

Supplement: S6 Fig — (a) EGFP-tagged Pip5k1c was coexpressed with FLAG-tagged MPP2 in HEK293T cells. EGFP-Pip5k1c was precipitated with αGFP antibody or normal IgG as negative control and analysed by western blot with αFLAG and αGFP antibodies. An additional IgG control lane is marked with an asterisk. (b) FLAG-tagged Farp1 was overexpressed together with EGFP-tagged MPP2 and copurifies with αGFP pull-down, as opposed to normal IgG as negative control. Co-immunoprecipitation was detected by western blot probing with αFLAG and αGFP antibodies. (c) Copurification of FLAG-tagged Ppp3ca (a Calcineurin subunit) overexpressed together with EGFP-MPP2 after αGFP pull-down or normal Ms IgG as negative control, detected by western blot with αFLAG and αGFP antibodies. (d) Co-immunoprecipitation of FLAG-tagged Arhgef2 together with EGFP-MPP2 after pull-down with αGFP antibody or IgG control, as detected by western blot using αFLAG and αGFP antibodies. (e) HA-tagged Gnao1 was overexpressed together with EGFP-tagged MPP2 in HEK293T cells. Upon pull-down with Ms αGFP antibody or normal Ms IgG, Gano1 copurification and GFP pull-down control were detected by western blot with αHA and αGFP antibodies. MPP2, membrane protein palmitoylated 2. (TIF) [file pbio.3001503.s006.tif]

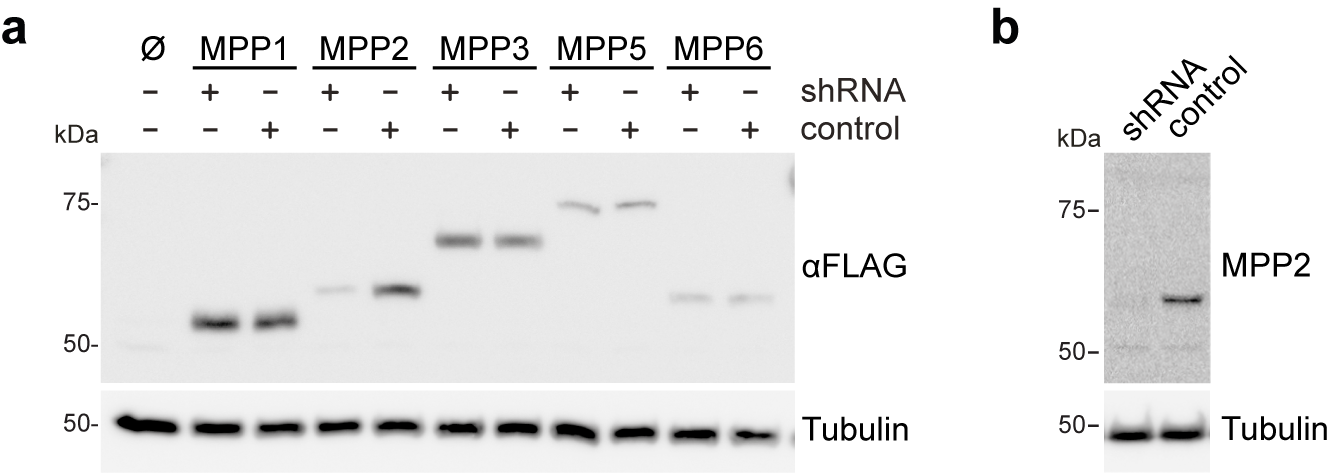

Supplement: S7 Fig — (a) Cotransfection in CHL V79 cells of MPP1, MPP2, MPP3, MPP5, and MPP6 expression constructs together with shRNA targeting MPP2 or control shRNA, respectively, leads to loss of expression of MPP2 together with shRNA, confirming efficacy and specificity of the selected shRNA sequence. (b) The same sequence introduced to cultured hippocampal neurons at DIV 3 with lentivirus-mediated knockdown, successfully abolishes expression of endogenous MPP2 as demonstrated by western blot analysis of whole cell lysates harvested at DIV 21 probed with aMPP2 antibody. DIV, days in vitro; MPP2, membrane protein palmitoylated 2. (TIF) [file pbio.3001503.s007.tif]

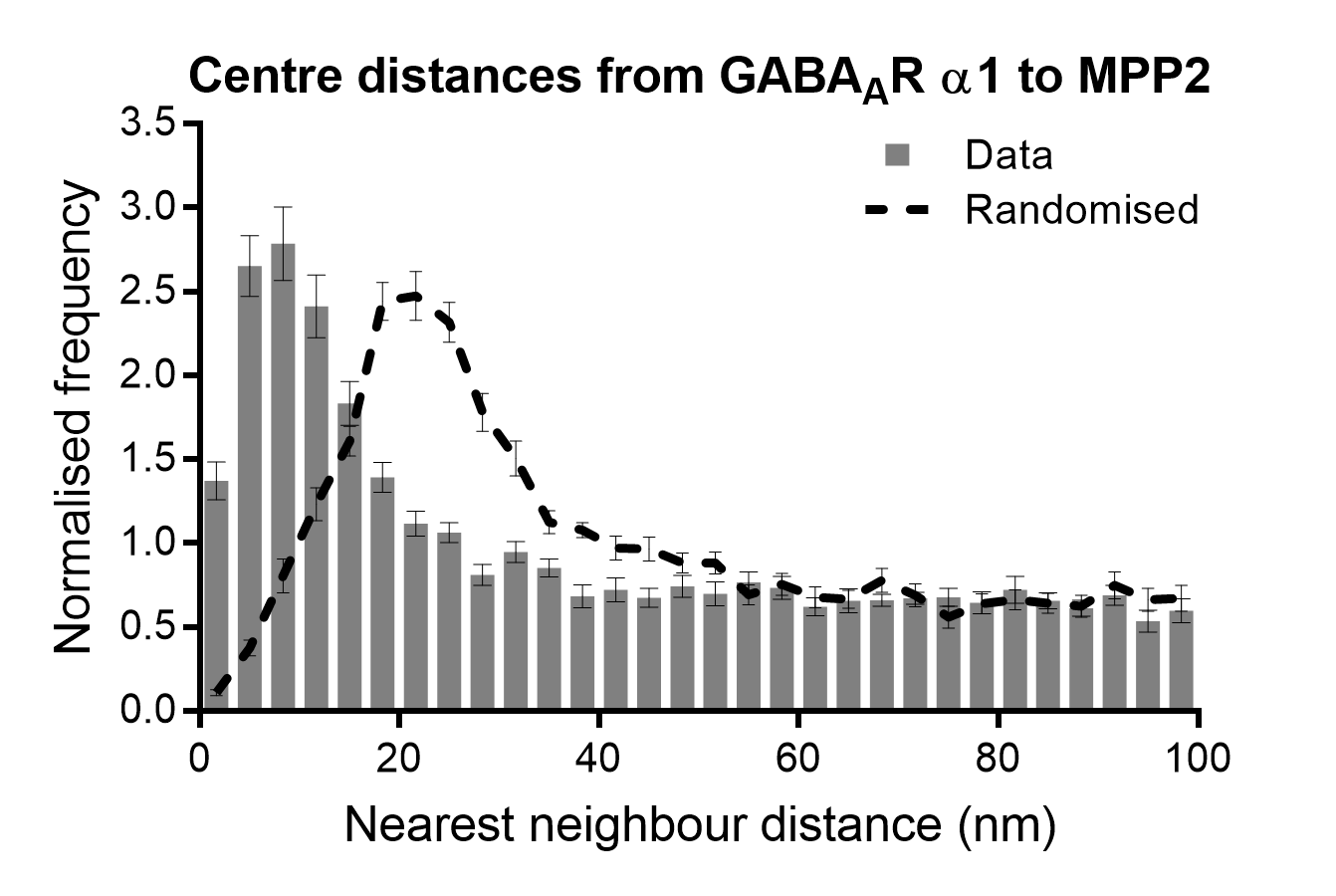

Supplement: S8 Fig — NN analysis of GABAAR α1 and MPP2 protein clusters after DBSCAN. NN distances were calculated from the cluster centres. Closest clusters of MPP2 to GABAAR α1 were analysed (grey bars). Dashed lines represent the random control by toroidal shift. Note the close association of both clusters (approximately 10 nm distance between both centres), which is well below the cluster sizes (20 and 40 nm, see Fig 6B) and shifted after randomisation. Mean ± SEM; n = 26 images from N = 3 independent experiments; dSTORM, direct stochastic optical reconstruction microscopy; GABA, γ-aminobutyric acid; MPP2, membrane protein palmitoylated 2; NN, nearest neighbour. (TIF) [file pbio.3001503.s008.tif]

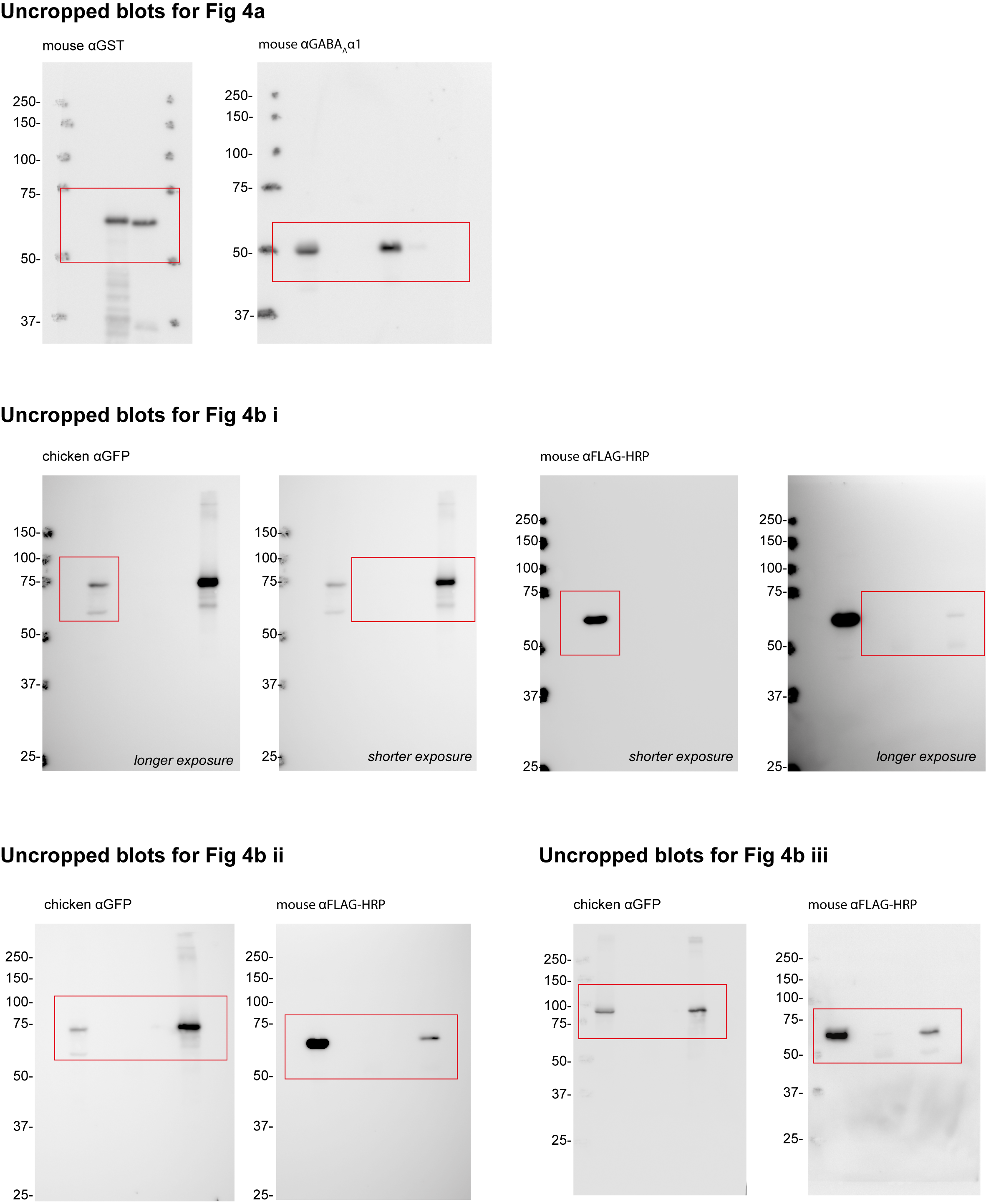

Supplement: S9 Fig — (TIF) [file pbio.3001503.s009.tif]

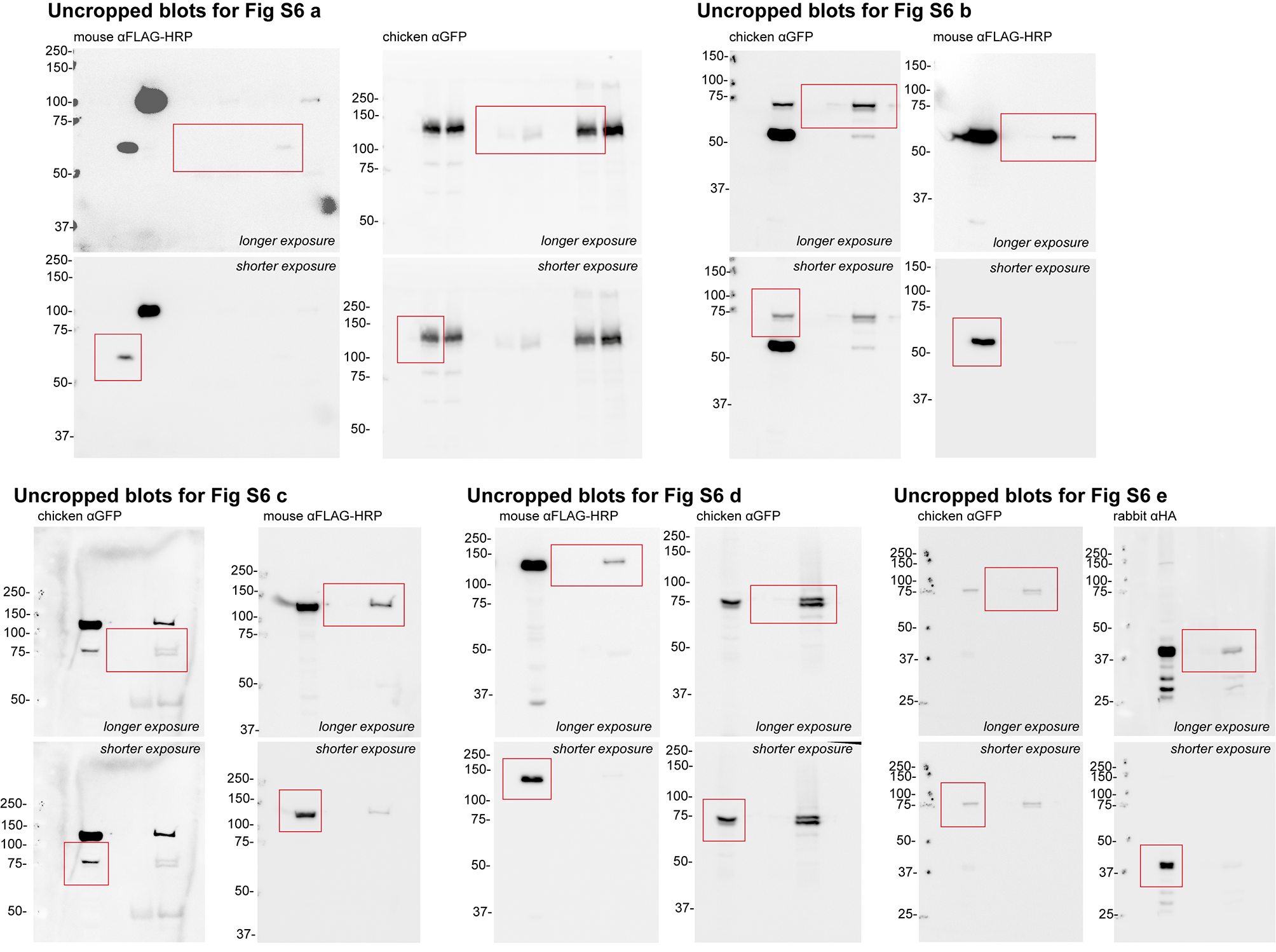

Supplement: S10 Fig — (TIF) [file pbio.3001503.s010.tif]

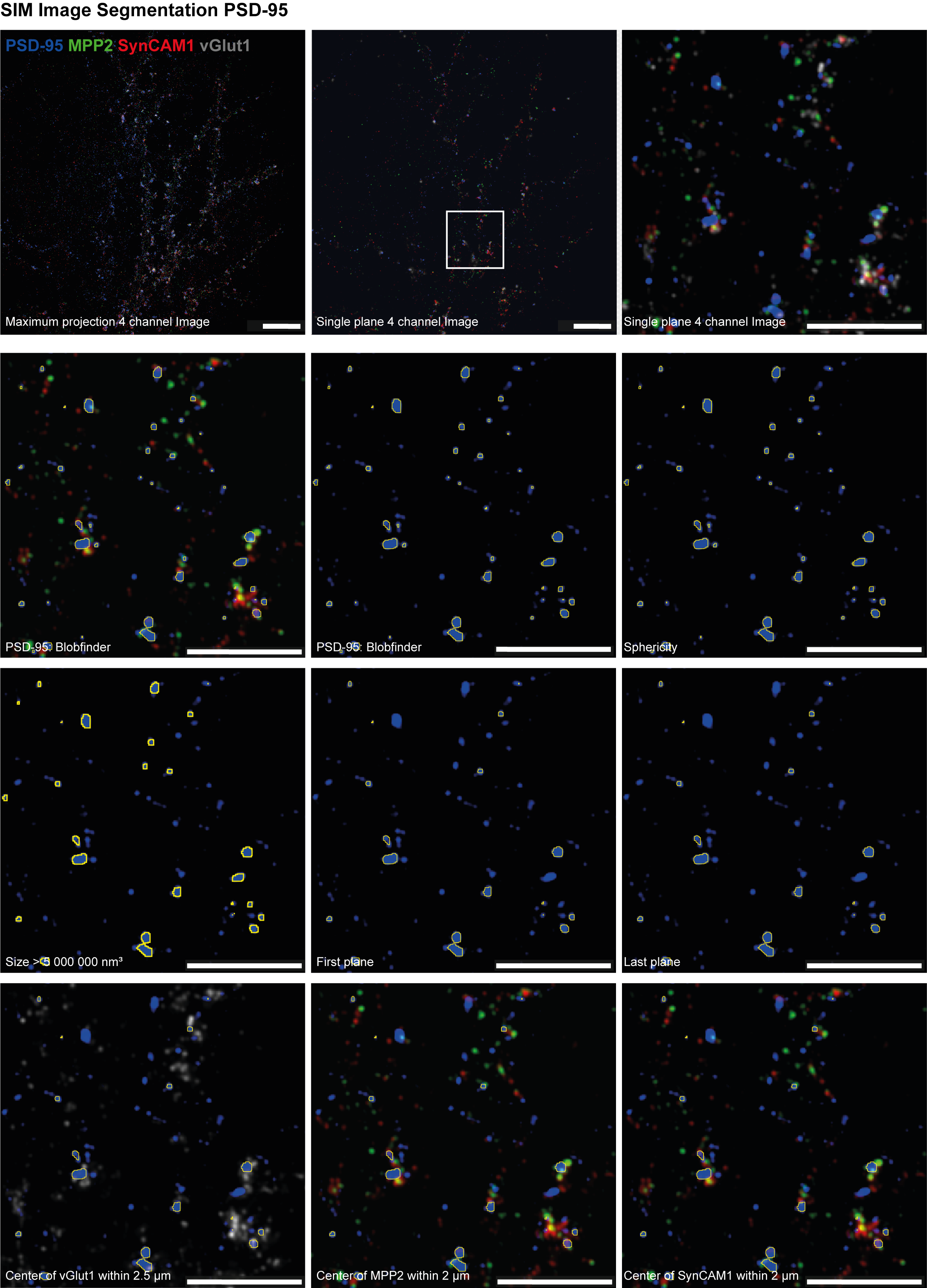

Supplement: S11 Fig — Image segmentation pipeline of PSD-95 signal implemented in Arivis Vision 4D. Top row left: maximum projected overview. Top row: single image plane overview. White box indicates location of detail view. Scale bar: 10 μm. Top right: single plane 4-colour detail view used to illustrate the segmentation steps below. Scale bar: 5 μm. PSD, postsynaptic density; SIM, structured illumination microscopy. (TIF) [file pbio.3001503.s011.tif]

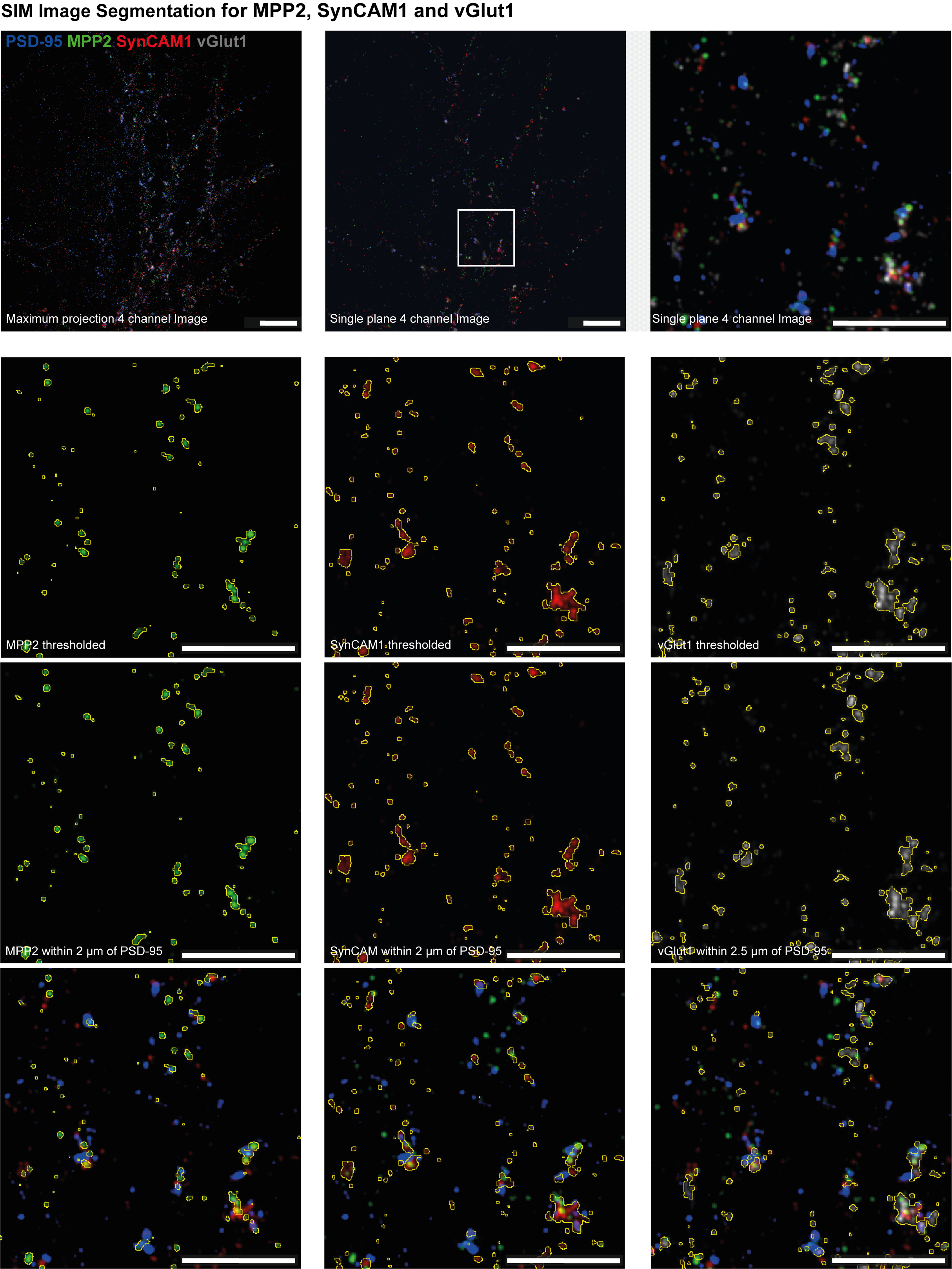

Supplement: S12 Fig — Image segmentation of MPP2, SynCAM 1, and vGlut1 signal implemented in Arivis Vision 4D. Top row left: maximum projected overview. Top row: single image plane overview. White box indicates location of detail view. Scale bar = 10 μm. Top right: single plane 4-colour detail view used to illustrate the segmentation steps below. Scale bar = 5 μm. Lower panel, left column: MPP2 segmentation; middle column: SynCAM 1 segmentation, right column: vGlut1 segmentation. Respective image channels were segmented by applying Otsu’s and Yen’s auto-threshold methods and selecting segments within 2 μm around a PSD-95 cluster (blue). MPP2, membrane protein palmitoylated 2;PSD, postsynaptic density; SIM, structured illumination microscopy. (TIF) [file pbio.3001503.s012.tif]

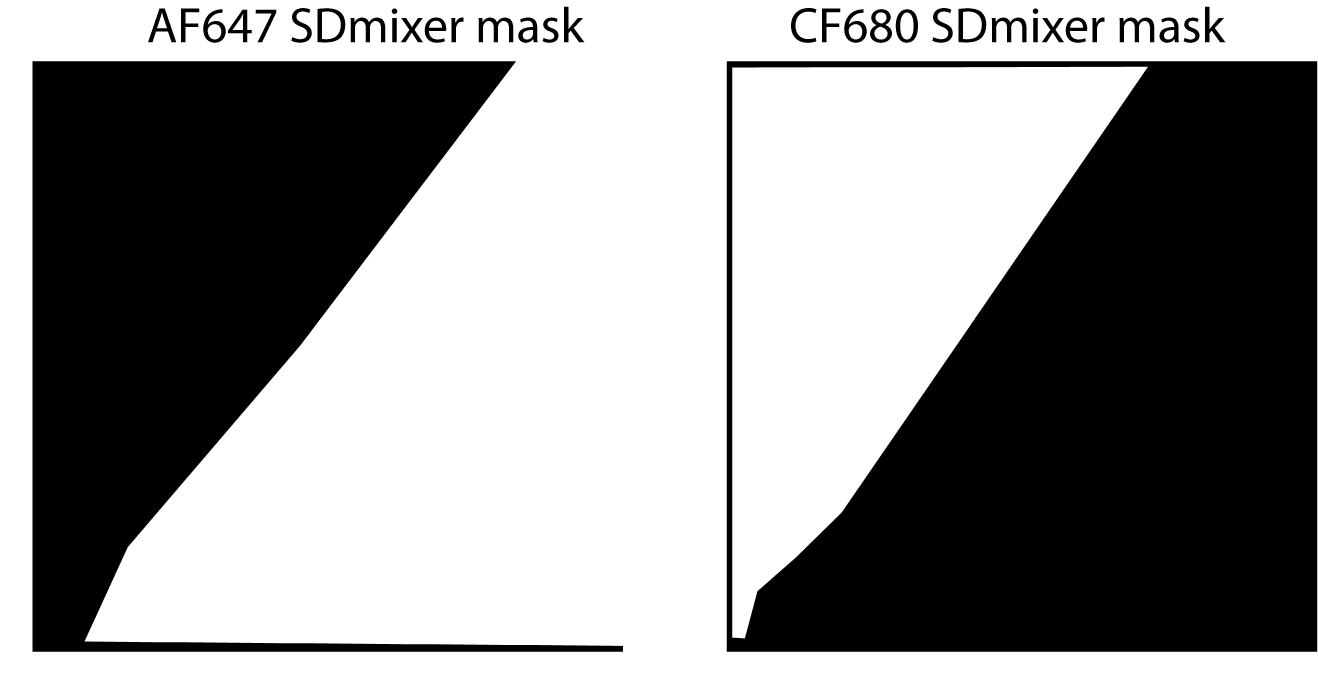

Supplement: S13 Fig — Colour separation masks for spectral de-mixing (SD)-dSTORM. dSTORM, direct stochastic optical reconstruction microscopy. (TIF) [file pbio.3001503.s013.tif]

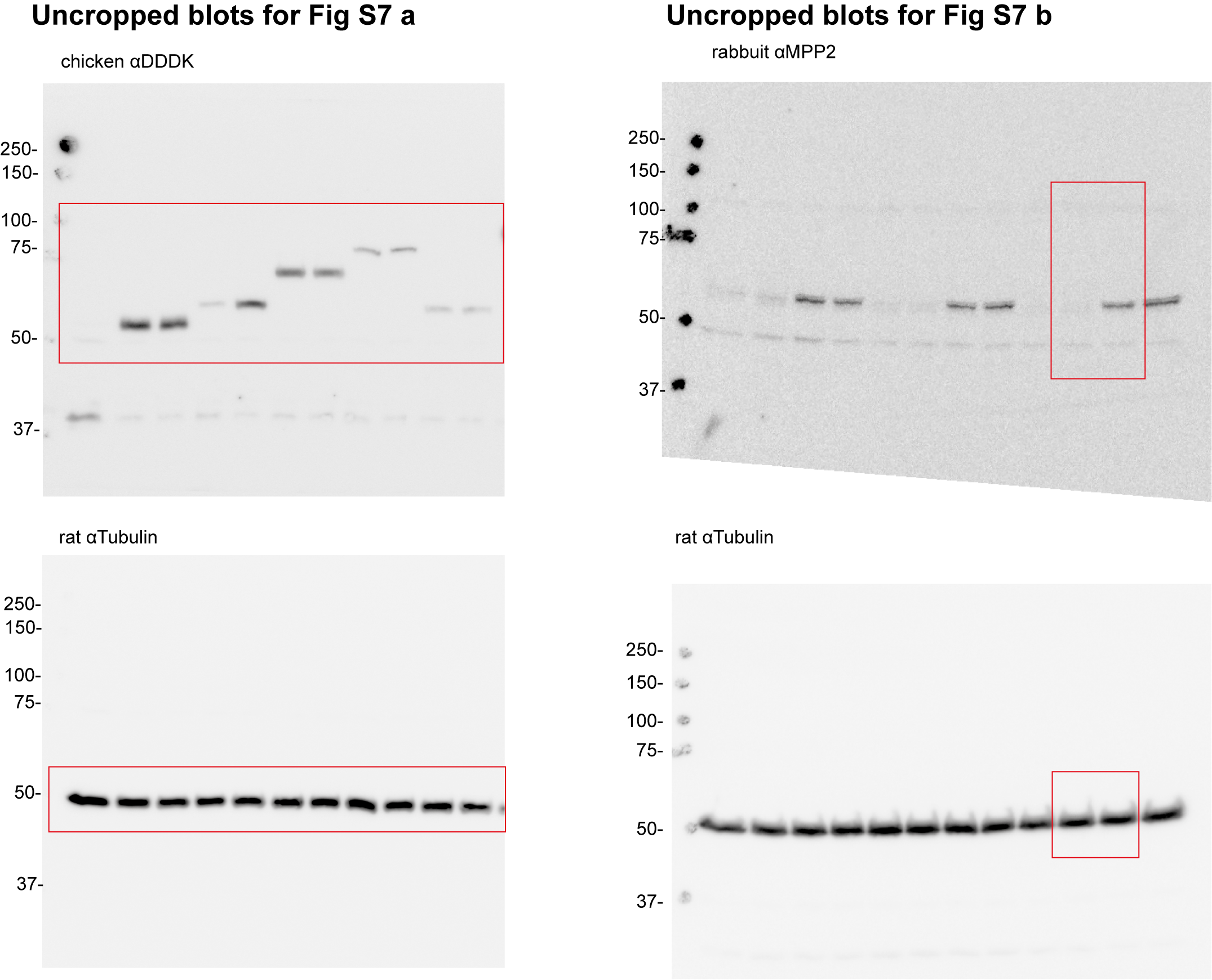

Supplement: S14 Fig — (TIF) [file pbio.3001503.s014.tif]
